# Geochemical fate of lead in contaminated residential soils following application of amendments for lead immobilization

**DOI:** 10.3389/fchem.2026.1742013

**Published:** 2026-02-05

**Authors:** Hadeer Saleh, Washington Braida, Zhiming Zhang, Rupali Datta, Dibyendu Sarkar

**Affiliations:** 1 Department of Civil, Environmental, and Ocean Engineering, Stevens Institute of Technology, Hoboken, NJ, United States; 2 Department of Civil and Environmental Engineering, Rowan University, Glassboro, NJ, United States; 3 Department of Biological Sciences, Michigan Technological University, Houghton, MI, United States

**Keywords:** geochemical fractionation, *in-situ* immobilization, lead contamination, soil amendments, soil remediation, urban soils

## Abstract

**Background:**

Lead (Pb) persists in urban soils, where its partitioning among geochemical fractions governs mobility, bioavailability, and human health risk. These fractions are strongly controlled by soil physicochemical properties, necessitating site-specific remediation strategies.

**Methods:**

This study developed a site-specific Pb immobilization framework linking amendment selection to soil geochemistry and sustainability considerations. Pb-contaminated residential soils from three U.S. cities, San Antonio (alkaline), Baltimore (acidic), and Detroit (near-neutral), were treated with gypsum, biochar + lime, and alum, respectively. Changes in Pb speciation were tracked using sequential extraction over 7, 30, and 90 days.

**Results:**

All amendments significantly reduced exchangeable Pb (F1) and increased less mobile fractions (F2–F3). Gypsum reduced F1 by ∼30% in San Antonio soils with minimal pH change, coincident with increased carbonate- and oxide-bound Pb. Biochar + lime reduced F1 by ∼50% in Baltimore soils, driven by a 0.4–0.8 pH increase and enhanced carbonate- and organic-bound Pb (F2–F4). Alum reduced F1 by ∼28% in Detroit soils, with transient pH shifts and strong increases in oxide-bound Pb (F3).

**Conclusion:**

Despite contrasting soil chemistries, all treatments achieved rapid and statistically significant Pb stabilization via distinct mechanisms, including Ca^2+^-facilitated precipitation, pH-driven surface complexation, and Al-hydroxide sorption. This work provides a mechanistic, transferable framework for tailoring low-cost, in situ amendments to local soil geochemistry to durably reduce Pb bioavailability and exposure risk in urban residential soils.

## 1 Introduction

Anthropogenic activities like mining, smelting, the use of leaded gasoline, lead-based paints, battery recycling, and industrial emissions within the past century have resulted in widespread Pb contamination of urban and residential soils (Al-Sabbagh, and Shreaz, 2025; Asare and Afriyie, 2021; Raj and Das, 2023). Lead is highly persistent and non-biodegradable, capable of long-term partitioning into different geochemical fractions of the soil, which ultimately controls its mobility, bioavailability, and risk to human health and the environment (Mousavi et al., 2022; Rammala et al., 2025). In soils, Pb can exist in multiple geochemical forms, which include soluble and exchangeable, carbonate-associated, oxide-bound, organic-bound, and silicate-bound forms (Tessier et al., 1979). The distribution across these fractions determines whether Pb is readily leachable and available for biological uptake or strongly immobilized and environmentally stable. Therefore, effective remediation approaches should not only reduce the total concentration of Pb but also transform labile Pb fractions into more stable, less bioavailable forms.

Pb mobility and human exposure potential are strongly governed by its distribution among operationally defined geochemical fractions, particularly the exchangeable (F1) pool, which represents the most labile and bioaccessible form. Recent studies have demonstrated strong positive correlations between F1-Pb and *in-vitro* bioaccessibility (IVBA) as well as *in-vivo* relative bioavailability, indicating that sequential extraction provides a meaningful proxy for exposure risk (Goncalves et al., 2023; Lake et al., 2021; Li et al., 2020). Li et al. (2020) showed that Pb present in exchangeable and carbonate-bound fractions exhibits significantly higher bioaccessibility compared to oxide- or residual-bound pools, while Goncalves et al. (2023) reported that a 30%–60% reduction in F1-Pb strongly decreased bioaccessible Pb in urban soils. These findings align with earlier evidence demonstrating that Pb redistribution from F1 into more stable phases markedly reduces the absorbed dose predicted by models such as IEUBK (U.S. EPA, 2007; Drexler and Brattin, 2007). Accordingly, soil amendments that shift Pb into carbonate-, oxide-, and residual-bound fractions are widely recognized as effective for reducing human exposure risk (Nie et al., 2024).

Traditional remediation strategies, such as excavation, soil washing, or replacement, are often prohibitively expensive, generate secondary wastes, disrupt soil structure, and are generally impractical for large urban areas (Priya et al., 2023; Samanth, 2024). Phytoextraction is also largely ineffective for Pb, given its poor mobility in soils and the limited uptake efficiency of most plants (Rahman et al., 2024; Tang, 2021). As a result, *in situ* immobilization using soil amendments has emerged as a more sustainable, cost-effective, and widely applicable strategy for addressing Pb pollution in urban top soils (Imran, 2024; Levei et al., 2025; Lwin et al., 2023). A range of amendments, including lime, gypsum, phosphate materials, and biochar, have been studied for their ability to immobilize Pb through precipitation, adsorption, ion exchange, surface complexation, and redox transformations (Udeigwe et al., 2011; Guo et al., 2023; Lwin et al., 2023; Mbasabire et al., 2025; Shruthi et al., 2024; Si-rui et al., 2021). The efficacy of these materials depends strongly on both soil characteristics, such as pH, cation exchange capacity, and organic matter content, and amendment properties, including surface area and chemical reactivity. Since these soil properties govern metal speciation, adsorption, and transformation mechanisms, their variability may lead to drastically different amendment performance across locations.

Despite such research, several critical knowledge gaps remain. Many studies either focus on the overall reduction of total Pb concentrations without tracing how geochemical fractionation changes during treatment or have emphasized bulk Pb stabilization rather than temporal redistribution into specific geochemical fractions. While ample studies on Pb immobilization in shooting range berms have indeed shown that phosphate, lime, and biochar amendments significantly reduce Pb mobility and leachability, a number of these studies (Basta et al., 2001; Cao et al., 2003; Khan et al., 2023) have generally focused on bulk Pb stabilization rather than temporal redistribution to specific geochemical fractions. The few comparative investigations conducted across geographically different soils, often with distinctly different baseline physicochemical properties, are rare; similarly, systematic examinations of the temporal dynamics of Pb redistribution among geochemical fractions are also rare.

This study addresses these gaps by investigating Pb-contaminated soils from three urban regions in the United States: San Antonio, TX; Baltimore, MD; and Detroit, MI. These cities were selected to represent distinct soil types and contamination histories that influence Pb geochemistry and amendment performance. In general, San Antonio soils are alkaline and calcareous, whereas Baltimore soils are moderately acidic (Yesilonis et al., 2008). In comparison, Detroit soils are finer-textured, with higher clay associated with legacy industrial emissions (Howard and Orlicki, 2015; Saleh et al., 2025). This variability allows for a comparative assessment of how amendment performance differs under contrasting pH, cation exchange capacity, and mineralogical conditions. To induce such changes, a suite of treatments was applied that included pistachio shell biochar, lime, gypsum, and alum. Changes in Pb fractionation were tracked over incubation periods of 7, 30, and 90 days. In this paper, changes in Pb fractionation induced by these amendments were evaluated using sequential extraction analysis combined with statistical testing to assess the extent to which treatments shift Pb from more labile to more stable fractions. Our findings elucidate soil-specific mechanisms controlling Pb immobilization, and sequential extraction and pH monitoring data highlight the distinct amendment–soil interactions that inform best practices for sustainable remediation strategies to reduce exposure risks from contaminated urban soils.

## 2 Materials and methods

### 2.1 Soil collection, preparation, and characterization

Residential soils were collected from ten houses each in Baltimore, San Antonio, and Detroit, U.S. At each site, a detailed grid survey of Pb concentrations was first performed using a handheld portable X-ray fluorescence analyzer (pXRF, Thermo Fisher Scientific, Niton XL3t) on 1.2 m^2^ grids. Based on the pXRF survey, samples were collected from identified Pb “hotspots” at a depth of 0–15 cm. Five subsamples were taken per housing unit, homogenized, and composited into a representative sample. In the laboratory, samples were air-dried and sieved through a 2 mm mesh prior to analysis.

Soils were characterized for texture, pH, electrical conductivity (EC), cation exchange capacity (CEC), soil organic matter (SOM), and major elements (Pb, Fe, Al, Ca, Mg, and P). Soil characterization followed the protocols outlined in the Soil Science Society of America Handbook for Chemical and Mineralogical Analysis (Sparks, 2020). SOM was quantified using the loss-on-ignition (LOI) method (Schulte and Hopkins, 1996). Total elemental concentrations were determined after acid digestion with HNO_3_ and H_2_O_2_ following USEPA Method 3050B (U.S. Environmental Protection Agency, 1996), with analysis by inductively coupled plasma–optical emission spectroscopy (ICP-OES, Agilent 5100). All analyses were conducted in triplicate. QA/QC protocols followed USEPA guidelines (Barth et al., 1989; U.S. Environmental Protection Agency, 1996; USEPA, 2023).

### 2.2 Amendment selection

The selection of soil amendments in this study was guided by our previously published work, which examined correlations between physicochemical properties and exchangeable Pb fractions across soils from San Antonio, Baltimore, and Detroit (Saleh et al., 2023). A dose of 3% (w/w) gypsum (CaSO_4_·2H_2_O; Alpha Chemical, Stoughton, MA, United States) was selected as a sustainable source of soluble Ca^2+^ to promote Pb precipitation and reduce Pb mobility in San Antonio soils. For Baltimore soils, which are moderately acidic, a combination of pistachio shell biochar (Persist™ biochar, V-Grid Energy Systems Inc., Camarillo, CA, United States, VGrid Energy Systems, 2025) at a dose of 5% (w/w) and lime [Ca(OH)_2_; Sigma-Aldrich, St. Louis, MO, United States] at a low dose (0.01% w/w) was used. In Detroit soils, alum [Al_2_(SO_4_)_3_·14H_2_O; Ferti-lome, Bonham, TX, United States] was selected as an amendment to promote Pb association with Al–organic complexes and oxide phases while reducing dissolved organic carbon. Alum was applied at 0.05% (w/w).

### 2.3 Incubation study design

Incubation experiments were conducted for 90 days at room temperature (25 °C), with samples collected at 0 (control), 7, 30, and 90 days. Soils were adjusted to 80% water-holding capacity and maintained at constant moisture in sealed polyethylene bags. For each city, 10 independent composite soil samples were collected from 10 different residential properties. Each composite sample was split into control and treated portions for incubation at each time interval (7, 30, and 90 days). Therefore, both pH and Pb fractionation analyses were based on n = 10 independent field samples per treatment per city per time point. Each composite sample was analyzed in triplicate, and the mean of the triplicates was used as the representative value for each property. The triplicates represent analytical precision, whereas the 10 property-level means represent statistically independent replicates used for treatment comparisons.

### 2.4 Sequential extraction and Pb speciation

Pb fractionation was determined by sequential extraction following Tessier et al. (1979), modified by Carbonell-Barrachina et al. (1999). The procedure partitioned Pb into five operationally defined fractions: (F1) soluble + exchangeable, (F2) carbonate-bound, (F3) Fe/Mn oxide-bound, (F4) organic matter-bound, and (F5) residual silicate-bound. A modified Tessier sequential extraction procedure was used, following Carbonell-Barrachina et al. (1999), in which the reducing step (F3) employs hydroxylamine hydrochloride under controlled heating to improve the dissolution of amorphous Fe/Mn oxides, and the oxidizing step (F4) uses hydrogen peroxide at adjusted pH to enhance recovery of organic-bound Pb. These modifications were adopted to increase extraction efficiency for soils with variable mineralogy and organic matter content. In brief:

F1: 1 g of soil was extracted with 8 mL 0.5 M Mg(NO_3_)_2_ (pH 7.0) at room temperature for 30 min. F2: The residue was treated with 8 mL 1 M NaOAc (pH 5.0).F3: The step-2 residue was heated with 20 mL 0.08 M NH_2_OH·HCl in 25% acetic acid (pH 5.0) at 96 °C for 6 h. F4: The step-3 residue was extracted with 3 mL 0.02 M HNO_3_ and 5 mL 30% H_2_O_2_ (pH 2.0), heated at 85 °C for 5 h, followed by the addition of 5 mL 3.2 M CH_3_COONH_4_ in 20% HNO_3_. F5: The final residue was digested with 25 mL concentrated HNO_3_ at 105 °C. After each step, suspensions were centrifuged at 3,500 × g for 20 min, and supernatants were filtered and analyzed for Pb by ICP-OES. Residues were used for subsequent steps.

Mass balance and recovery were assessed by summing Pb across the five operationally defined fractions and comparing the total to bulk Pb concentrations obtained from acid digestion. Recoveries ranged from 92% to 103%, indicating minimal loss or contamination across steps.

### 2.5 Life cycle analysis (LCA)

A preliminary life cycle analysis (LCA) was performed to evaluate the environmental sustainability of the amendment-based Pb immobilization strategies. The assessment followed the ReCiPe 2016 Midpoint methodology and included the following steps:

Goal and Scope Definition: The goal was to quantify the cradle-to-application environmental impacts associated with immobilizing Pb in contaminated garden soils using the selected amendments, pistachio shell biochar + lime, alum, and gypsum, applied as a single field treatment. The functional unit was defined as the treatment of 1 m^2^ of Pb-contaminated soil to a depth of 30 cm, assuming a bulk density of 1,500 kg m^-3^ (450 kg of soil). The system boundary encompassed amendment production, transportation, and field application. All transportation was modeled as diesel truck freight using GREET (2022) emission factors.

Inventory and Impact Assessment: Life-cycle inventories were developed for each amendment–soil combination described in Section 2.2, accounting for material inputs, energy use, and emissions throughout the defined boundary. Environmental impacts were quantified using the ReCiPe 2016 Midpoint approach, focusing on impact categories such as global warming potential and resource depletion (Huijbregts et al., 2017). The inventory analysis for the preliminary LCA of Pb immobilization treatments in the studied soils is summarized in Supplementary Tables S16–S21. Transportation distances for the amendments were estimated based on the procurement site location and the city, as described in the Supplementary Material.

**FIGURE 1:**
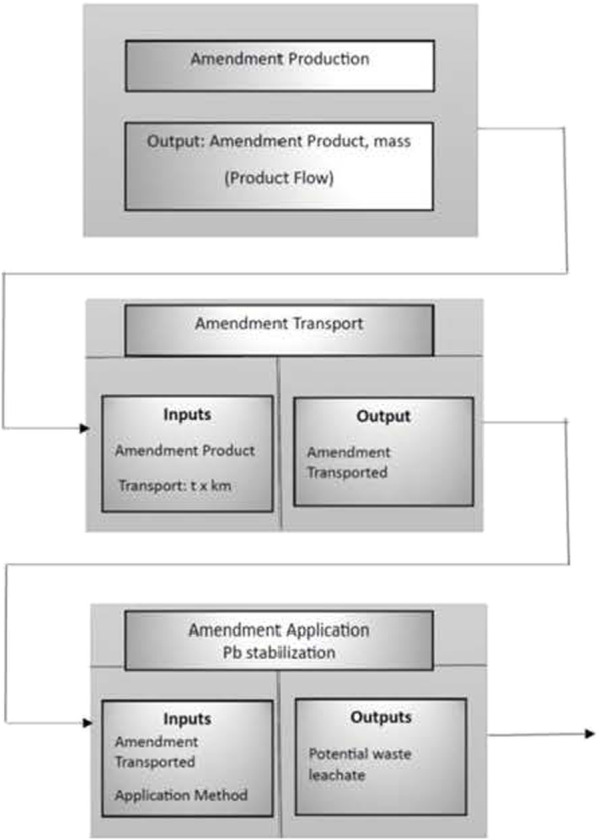
LCA model for Pb immobilization in soil.

Figure 1 shows the LCA model for Pb immobilization in soil. Moreover, a comparison of the environmental impacts of the proposed immobilization approaches vs. soil excavation and landfilling, assuming a 50-mile distance to the landfill, was performed.

### 2.6 Statistical analyses

All statistical analyses were conducted to evaluate the effects of amendment treatments on pH and Pb geochemical fractionation at each incubation period (7, 30, and 90 days). For each city, n = 10 independent composite soil samples (one per property) were used per treatment and time point. Each sample was analyzed in triplicate, and the triplicate mean was treated as the unit of observation. Mean values and standard deviations (SD) were calculated for each Pb fraction (F1–F5) to assess variability within replicates. Prior to inferential testing, data normality was evaluated using the Shapiro–Wilk test. Differences between control and treated samples for each fraction and incubation period were analyzed using Welch’s two-tailed *t*-test, which does not assume equal variances between groups. Statistical significance was accepted at *p* < 0.05. For overall comparison of Pb redistribution among fractions and time points within each soil type, a one-way analysis of variance (ANOVA) followed by Tukey’s honest significant difference (HSD) *post hoc* test was additionally performed to identify pairwise differences (*p* < 0.05). All statistical computations were performed using JMP 14 (SAS Institute Inc., Cary, NC, United States) and Microsoft Excel (2018).

## 3 Results and discussion

### 3.1 Physicochemical properties of soils and implications for Pb behavior

Baseline soil characterization was published in an earlier article (Saleh et al., 2023). Distinct physicochemical contrasts were observed among the three study sites that control Pb speciation and mobility, which provided the rationale behind amendment selection (Supplementary Tables S1–S3).

San Antonio soils were neutral to alkaline (pH 7.4–7.9) with elevated Ca (20,650–72,141 mg kg^-1^), moderate-to-high SOM (5.2%–12.9%), and variable clay content (7%–65%). Pb concentrations ranged widely (361–7,768 mg kg^-1^). The combination of high Ca and alkaline pH favors carbonate precipitation and Ca–Pb interactions that stabilize Pb in less mobile forms, consistent with previous findings in Ca-rich soils (Haque et al., 2021; Khedr et al., 2023; Landrot and Khaokaew, 2020). The exchangeable Pb fraction in San Antonio soils showed significant negative correlations with total Ca (r = −0.438) and Al (r = −0.653), suggesting that Ca-based amendments could effectively stabilize Pb in these alkaline soils. Consequently, gypsum was chosen as the amendment for these soils.

Baltimore soils were acidic (pH 5.2–6.4) with low clay (2.1%–5.2%), low CEC (5–9 meq 100 g^-1^), and minimal SOM (0.6%–2.5%). Total Pb concentrations (39–5,373 mg kg^-1^) were lower than in San Antonio, yet the low pH and limited sorption capacity increase Pb solubility and exchangeable pools (Chamorro and Sánchez-Andica, 2024; Kicińska et al., 2022). Elevated Al (22,000–31,000 mg kg^-1^) and Fe (14,000–21,000 mg kg^-1^) contents provide potential oxide sorption sites, but their stabilizing effect is highly pH-dependent. Hence, a combination of pistachio shell biochar and lime was selected as the amendment. Pistachio shell biochar used was produced by high-temperature gasification (>1,100 °C), contains >80–90% carbon, low ash content, and high surface area, providing abundant sorption sites and phosphate enrichment (Table 1). Lime was added at a low dose alongside biochar to enhance pH buffering capacity and facilitate carbonate precipitation, thereby addressing soil acidity and improving Pb immobilization.

**TABLE 1:** Physicochemical properties of soil amendments used in the incubation experiments.

Amendment	pH	EC (µS cm^-1^)	Major elements (mg kg^-1^)	BET surface area (m^2^ g^-1^)
Pistachio shell biochar	8.8	645	Ca: 12,350; Mg: 2,110; K: 7,520; P: 1,030; Fe: 1,420	149.2
Lime (Ca(OH)_2_)	12.4	1,210	Ca: 365,000; Mg: 2,800; Fe: 520	-
Gypsum (CaSO_4_·2H_2_O)	7.2	1,480	Ca: 232,000; S: 185,000; Mg: 1,210	-
Alum (Al_2_(SO_4_)_3_·14H_2_O)	3.1	1,820	Al: 148,000; S: 215,000; Fe: 740	-

Detroit soils were near-neutral to slightly alkaline (pH 6.3–7.9) with moderate clay (6.8%–20%), relatively high CEC (11.6–64.3 meq 100 g^-1^), and higher SOM (4.7%–10.4%) than Baltimore. Pb concentrations were moderate (124–1,575 mg kg^-1^). High Al and Fe levels (33,600–38,200 mg kg^-1^ and 4,400–15,600 mg kg^-1^, respectively) favor Pb binding to Fe/Al-oxide and organic ligands, particularly under buffered pH conditions (Cui et al., 2024; Noerpel et al., 2025; Steinberg and Hodge, 2022). In Detroit soils, the exchangeable Pb fraction was significantly negatively correlated with total Al (r = −0.628) and SOM (r = −0.408). Given the high organic matter content of these soils, alum was selected as an amendment to promote Pb association with Al–organic complexes and oxide phases while reducing dissolved organic carbon.

These geochemical fractionation patterns of Pb in the soils from the three cities are supported by sequential extraction data from Saleh et al. (2023). While San Antonio soils were dominated by carbonate- and organic-bound Pb, Baltimore soils by exchangeable and oxide-bound Pb, and Detroit soils by organic- and oxide-bound Pb. Correlation analysis further showed negative associations between exchangeable Pb and Ca (San Antonio), pH and P (Baltimore), and Al and SOM (Detroit). Collectively, these contrasts guided the amendment strategy: gypsum for Ca-rich alkaline soils to enhance carbonate stabilization (San Antonio), biochar + lime to raise pH and promote organic/carbonate binding in acidic soils (Baltimore), and alum to exploit Al-hydroxide sorption in near-neutral soils (Detroit). The physico-chemical properties of the amendments are shown in Table 1. The following Section 3.2 evaluates the amendment-induced redistribution of Pb among geochemical fractions across incubation periods.

In this study, Pb is classified as a “major measured element” strictly in the analytical sense because its concentrations (hundreds–thousands mg/kg) were orders of magnitude higher than other trace metals, requiring calibration within the major-element detection range; this designation does not imply percent-level geological abundance in residential soils.

### 3.2 Mechanistic interpretation of Pb immobilization in San Antonio soils with gypsum

#### 3.2.1 pH dynamics

**FIGURE 2:**
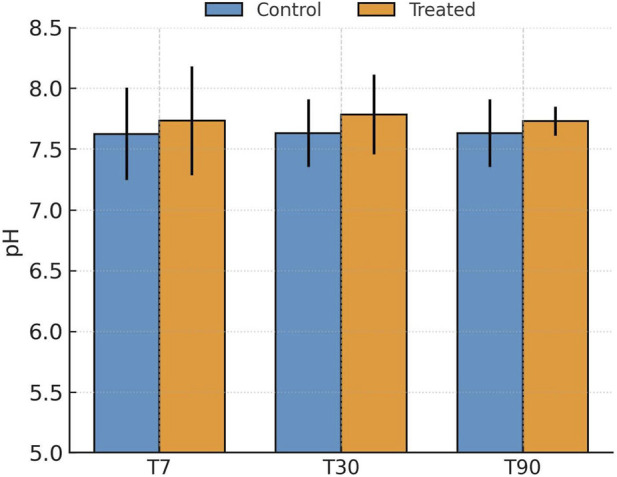
Evolution of soil pH (mean ± SD, n = 10 independent composite soil samples per city) in control and gypsum-amended San Antonio soils after 7, 30, and 90 days of incubation.

Gypsum application produced a modest yet consistent increase in soil pH throughout the incubation period (T7–T90, where T7, T30, and T90 refer to samples collected after 7, 30, and 90 days of incubation) (Figure 2). San Antonio soils were initially slightly alkaline (pH 7.4–7.8), and gypsum addition elevated pH by approximately 0.1–0.2 units (Supplementary Table S4). Even minor increases in pH can reduce Pb solubility by enhancing hydroxyl ion activity and promoting carbonate equilibria. Following gypsum application, elevated Ca^2+^ availability is expected from gypsum dissolution under alkaline conditions, which enhances cation exchange and carbonate equilibria (Haque et al., 2021; Khedr et al., 2023). This Ca^2+^ enrichment likely drove the observed decline in exchangeable Pb (F1) and the concurrent increase in carbonate-bound Pb (F2), consistent with Ca-mediated precipitation and ion exchange mechanisms, as described in Section 3.2.3. These modest but persistent pH and ionic changes created a favorable geochemical environment for Pb immobilization.

#### 3.2.2 Redistribution of Pb fractions

**FIGURE 3:**
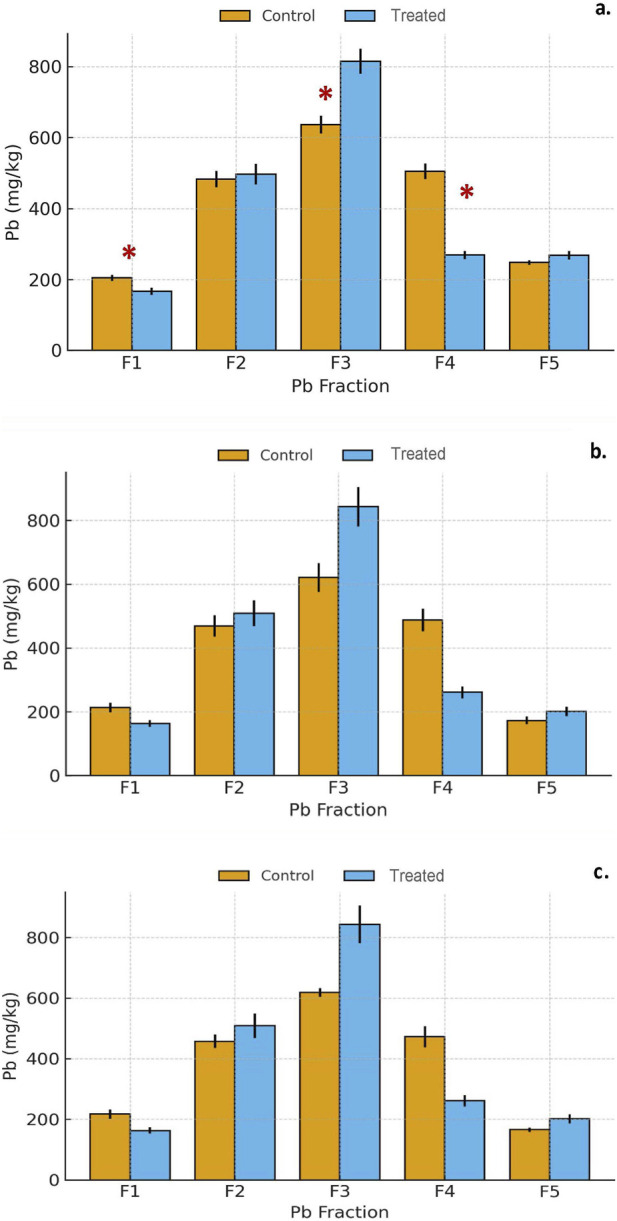
**(a–c)** Distribution of Pb among geochemical fractions (F1–F5) in San Antonio soils after 7, 30, and 90 days of incubation. Bars represent the mean ± standard deviation (n = 10 independent composite soil samples per city) for control and treated groups. Each value corresponds to the mean of triplicate extractions per sample. Asterisks indicate significant differences between control and treated soils according to Welch’s t-test (*p* < 0.05).

Sequential extraction results revealed systematic and significant redistributions of Pb among operationally-defined fractions following gypsum treatment (Figures 3a–c; Supplementary Tables S7-S9).

Exchangeable (F1): Exchangeable Pb decreased markedly (∼25–30%) within the first 7 days (*p* = 0.0094) and continued to decline through 90 days of incubation (*p* = 0.0044). This sharp reduction reflects the displacement of Pb^2+^ from exchange sites by abundant Ca^2+^ ions released from gypsum dissolution. The elevated Ca^2+^ activity under alkaline conditions effectively promoted cation exchange, reducing Pb mobility in the most labile fraction. Carbonate-bound (F2): The F2 fraction exhibited a moderate increase (+6–8%) over time, consistent with gradual Pb incorporation into carbonate minerals such as cerussite (PbCO_3_) or co-precipitates with CaCO_3_. Gypsum dissolution enhanced the Ca^2+^ supply and shifted carbonate equilibria toward Pb–Ca carbonate formation. Similar Ca-mediated stabilization under alkaline environments has been documented by Landrot and Khaokaew (2020) and Lwin et al. (2023). Oxide-bound (F3): Pb associated with Fe/Mn oxides increased substantially (+30–35%) between 30 and 90 days, suggesting secondary stabilization via adsorption and co-precipitation with hydrous oxides (Rotter et al., 2017). This transformation indicates that gypsum indirectly facilitated Pb immobilization by promoting conditions that favored oxide-bound Pb phases. Organic matter-bound (F4): Pb associated with organic matter decreased significantly (∼50%), reflecting partial desorption or mineral association of organic-bound Pb under alkaline conditions. The loss of organic-bound Pb likely corresponded to redistribution into more stable carbonate and oxide-bound pools. Residual/silicate-bound (F5): The residual fraction increased slightly (∼7%), suggesting limited incorporation into mineral lattices within the short-term incubation. Overall, gypsum primarily influenced the more reactive pools (F1–F3), resulting in net stabilization of Pb through multi-pathway immobilization involving carbonate and oxide transformations.

#### 3.2.3 Gypsum: Calcium-driven Pb immobilization pathways

The fractionation trends (F1 ↓, F2 ↑, F3 ↑, F4 ↓, F5 ↑) show that gypsum immobilized Pb through Ca-driven reactions that strengthened with time. At T7, Ca^2+^ released from gypsum displaced Pb^2+^ from exchange sites, causing the rapid decline in F1 and initiating Pb incorporation into carbonate-associated forms (F2). This exchange process (Pb–X + Ca^2+^ ⇌ Ca–X + Pb^2+^) occurs readily in Ca-rich alkaline environments because Ca^2+^ has a stronger affinity for soil colloids and Pb^2+^ exhibits lower hydration energy under these conditions (Haque et al., 2021; Illera et al., 2004; Li et al., 2014).

By T30, continued Ca^2+^ availability and stable pH promoted further carbonate precipitation and strengthened Pb retention in F2, while increases in F3 reflected secondary stabilization on Fe/Mn-oxide surfaces via adsorption and co-precipitation. Spectroscopic evidence confirms that Pb binds to Fe-oxides through inner-sphere complexation, indicating strong Pb–O–Fe bonding rather than simple electrostatic attraction (O’Reilly and Hochella, 2003; Trivedi et al., 2003).

By T90, Pb was predominantly redistributed into carbonate- and oxide-bound fractions, accompanied by a decline in organic-bound Pb (F4) as Pb–organic complexes transitioned into more stable inorganic pools. Overall, gypsum promoted Pb stabilization through Ca-enhanced cation exchange followed by incorporation into carbonate and oxide phases, producing durable immobilization in alkaline soils.

### 3.3 Mechanistic interpretation of Pb immobilization in Baltimore soils with biochar + lime

#### 3.3.1 pH dynamics

**FIGURE 4:**
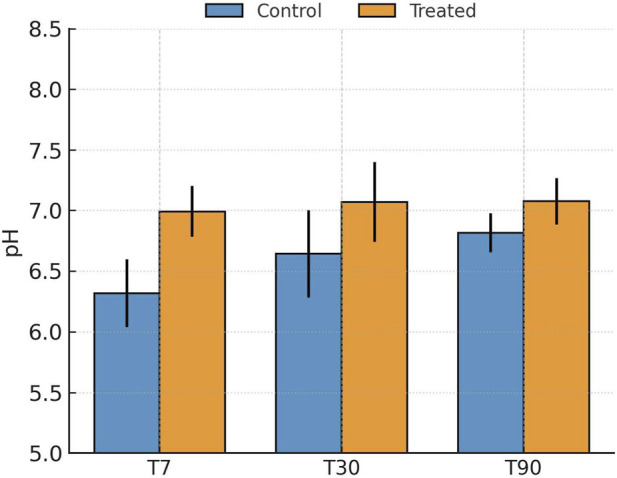
Evolution of soil pH (mean ± SD, n = 10 independent composite soil samples per city) in control and biochar + lime-amended Baltimore soils after 7, 30, and 90 days of incubation.

Baltimore soils, initially acidic (pH 5.1–6.4), responded strongly to the biochar + lime amendment, exhibiting rapid and sustained pH increases throughout incubation (T7–T90) (Figure 4). The pH rose by approximately 0.4–0.8 units within the first 7 days and stabilized near-neutral (6.8–7.3) conditions by day 90 (Supplementary Table S5). This shift was essential in altering Pb speciation, as higher pH reduces Pb^2+^ solubility, enhances surface negative charge on soil colloids, and drives precipitation of Pb carbonates and hydroxides. The alkaline ash component of biochar and the buffering capacity of lime acted synergistically to neutralize soil acidity, creating favorable conditions for Pb immobilization through multiple concurrent mechanisms (Ahmed et al., 2022; Cui et al., 2024).

#### 3.3.2 Redistribution of Pb fractions

**FIGURE 5:**
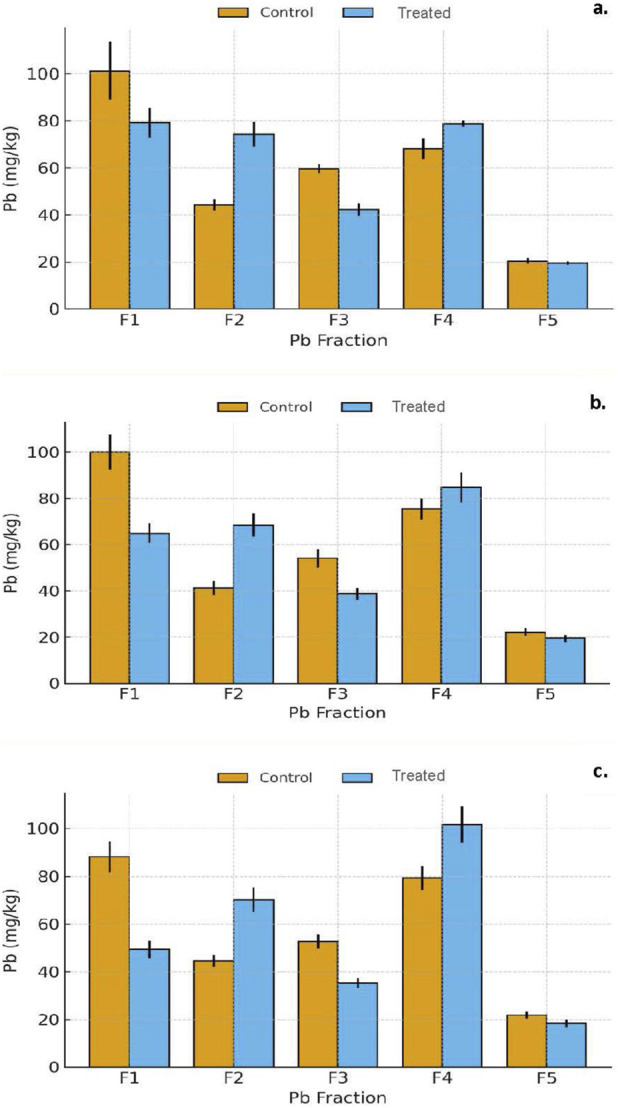
**(a–c)** Distribution of Pb among geochemical fractions (F1–F5) in Baltimore soils after 7, 30, and 90 days of incubation. Bars represent the mean ± standard deviation (n = 10 independent composite soil samples per city) for control and treated groups. Each value corresponds to the mean of triplicate extractions per sample. Asterisks indicate significant differences between control and treated soils according to Welch’s t-test (*p* < 0.05).

Combined biochar–lime addition caused significant and consistent redistributions of Pb among fractions, demonstrating the chemical stabilization of Pb into less labile pools (Figures 5a–c; Supplementary Tables S10-S12).

Exchangeable (F1): Exchangeable Pb declined sharply across all samples, dropping by roughly 40%–45% within 7 days (*p* = 0.0067) and up to 55% by 90 days (*p* = 0.01). This rapid reduction reflects both the strong sorption of Pb^2+^ onto negatively charged biochar surfaces and decreased Pb activity under elevated pH. The immediate fall in F1 indicates early-stage stabilization dominated by pH-induced deprotonation of biochar functional groups and cation exchange with Ca^2+^ released from lime. Carbonate-bound (F2): The F2 fraction increased substantially (+30–40%), particularly between T30 and T90, confirming progressive Pb incorporation into cerussite- and hydrocerussite-type carbonate phases. Sustained alkalinity and carbonate ion availability from lime enhanced co-precipitation of Pb with CaCO_3_. These findings confirm the synergy between lime and biochar in promoting stable Pb carbonate formation. Oxide-bound (F3): Pb associated with Fe/Mn oxides exhibited a slight decline (≈20%), suggesting that most Pb immobilization occurred via organic and carbonate pathways rather than oxide sorption. Nonetheless, minor retention on oxide surfaces may still contribute to secondary stabilization under near-neutral pH. Organic matter-bound (F4): A consistent increase of ≈25% in F4 was observed, driven by biochar’s large surface area and abundance of carboxyl, hydroxyl, and phenolic groups capable of forming strong Pb–organic complexes. Enhanced SOM stability under higher pH further reinforced these interactions. This pattern aligns with prior studies showing synergistic Pb immobilization via pH elevation and organic surface complexation (Noerpel et al., 2025; Steinberg and Hodge, 2022). Residual/silicate-bound (F5): The F5 fraction remained nearly stable (≤3% increase), indicating that Pb redistribution was confined to the more reactive pools (F1–F4) rather than incorporation into crystalline minerals.

#### 3.3.3 Biochar + lime: pH-driven immobilization and organic surface complexation

The fractionation patterns (F1 ↓, F2 ↑, F4 ↑, slight F3 ↓, stable F5) show that the combined biochar + lime amendment immobilized Pb through pH-driven geochemical shifts and enhanced sorption to organic surfaces. At T7, lime rapidly elevated soil pH from moderately acidic to near-neutral, decreasing Pb^2+^ solubility and increasing the negative charge on soil colloids and biochar surfaces. This shift produced an immediate decline in exchangeable Pb (F1) and a corresponding increase in organic-bound Pb (F4), reflecting strong complexation with carboxyl, hydroxyl, and phenolic functional groups (Igalavithana et al., 2019).

By T30, sustained alkalinity increased carbonate saturation, promoting PbCO_3_ precipitation and growth of the carbonate-bound fraction (F2). Organic-bound Pb remained elevated as biochar functional groups stayed deprotonated under higher pH, supporting stable organo-Pb associations (Hamid et al., 2020). The slight decline in oxide-bound Pb (F3) suggests that in these acidic soils, carbonate and organic complexation dominated Pb retention pathways.

By T90, Pb was predominantly partitioned into the carbonate- and organic-bound fractions, indicating durable stabilization through combined pH correction and biochar-mediated sorption (Palansooriya et al., 2022). These trends highlight that biochar + lime is particularly effective in acidic Baltimore soils, where pH strongly governs Pb speciation and mobility.

No additional treatment was included in which soils were amended with an inert, non-reactive solid (e.g., washed silica sand) at the same mass fraction as the reactive amendments. Thus, the experimental design isolates chemical effects relative to an unamended control but does not fully eliminate the possibility of a small physical dilution effect associated with the added solid mass. However, given the low amendment rates (≤5% w/w) and the near-quantitative Pb recoveries across sequential extraction fractions, any purely dilution-driven change in bulk Pb concentration is expected to be minor relative to the significant shifts observed in labile and carbonate/oxide-bound fractions.

### 3.4 Mechanistic interpretation of Pb immobilization in Detroit soils with alum

#### 3.4.1 pH dynamics

**FIGURE 6:**
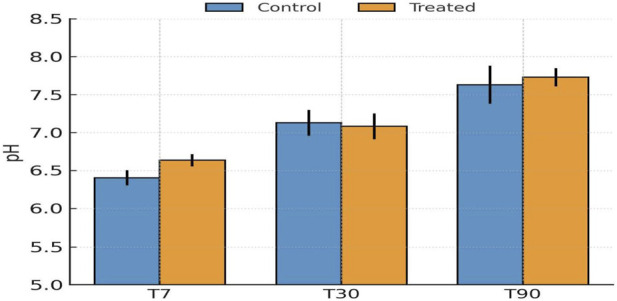
Evolution of soil pH (mean ± SD, n = 10 independent composite soil samples per city) in control and alum-amended Detroit soils after 7, 30, and 90 days of incubation.

Unlike the alkaline San Antonio or acidic Baltimore soils, Detroit soils were initially near-neutral (6.2–7.8) with moderate CEC and SOM (4.7%–10.4%), providing a balanced matrix for reactive Al species to interact with Pb (Figure 6). Following alum addition, pH declined slightly by 0.2–0.4 units during the first 7–30 days due to hydrolysis of Al^3+^ releasing H^+^ ions, stabilizing between 6.8 and 7.1 by day 90 (Supplementary Table S6). Despite the mild acidification, Pb immobilization was strong, indicating that the alum’s reactivity and the formation of Al-hydroxide phases offset the potential for increased Pb solubility. Similar behavior has been reported in alum-treated soils and sediments, where Al hydrolysis forms amorphous Al(OH)_3_ or Al–O–Pb complexes that immobilize heavy metals effectively (Lwin et al., 2023; Ouhadi and Goodarzi, 2006; Zhou and Haynes, 2011; Gillespie et al., 2021).

#### 3.4.2 Redistribution of Pb fractions

**FIGURE 7:**
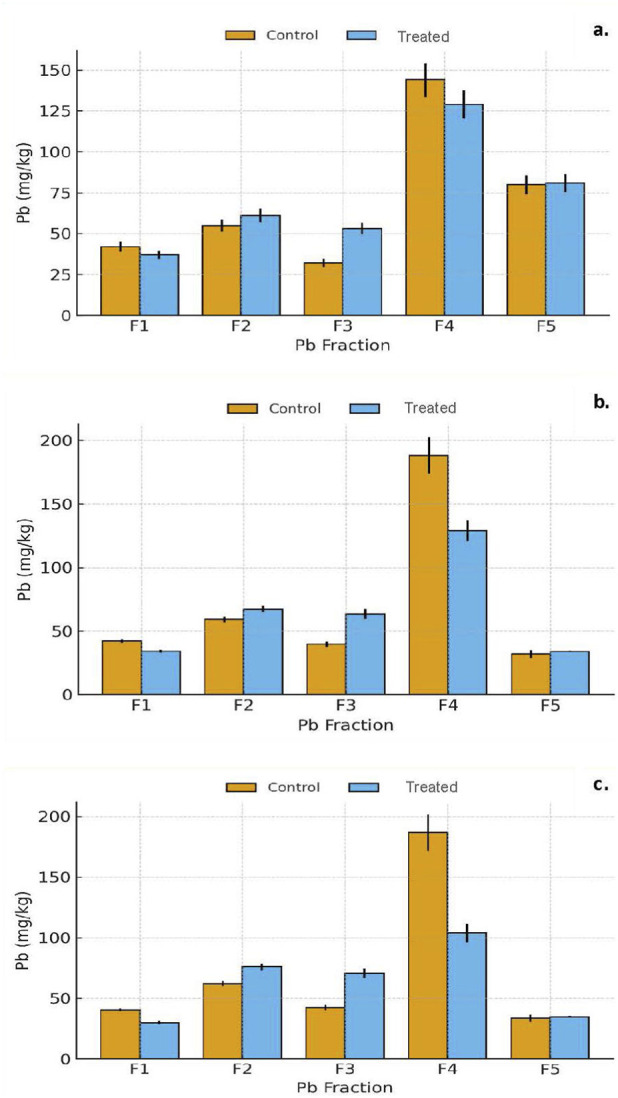
**(a–c)** Distribution of Pb among geochemical fractions (F1–F5) in Detroit soils after 7, 30, and 90 days of incubation. Bars represent the mean ± standard deviation (n = 10 independent composite soil samples per city) for control and treated groups. Each value corresponds to the mean of triplicate extractions per sample. Asterisks indicate significant differences between control and treated soils according to Welch’s t-test (*p* < 0.05).

Alum addition induced distinct and progressive shifts in Pb distribution across fractions, characterized by strong reductions in labile pools (F1) and enhanced stabilization in oxide and carbonate fractions (Figures 7a–c; Supplementary Tables S13-S15).

Exchangeable (F1): Exchangeable Pb decreased sharply within the first week (–35%–45%) (*p* = 1.7 × 10^−5^) and remained suppressed through day 90 (–50%–55%) (*p* = 1.8 × 10^−5^). This persistent decline reflects rapid Pb sorption onto freshly precipitated Al(OH)_3_ surfaces formed during alum hydrolysis and reduced competition from other cations. The effect was statistically significant across nearly all samples (p < 0.05), confirming durable immobilization of labile Pb. Carbonate-bound (F2): F2 increased moderately (+10–15%) throughout the incubation period, indicating that localized buffering and carbonate equilibria were maintained despite slight pH declines. The presence of Ca^2+^ and Mg^2+^ in the soil matrix likely facilitated Pb incorporation into mixed carbonate phases such as hydrocerussite (Pb_3_(CO_3_)_2_(OH)_2_) and Ca–Pb–CO_3_ solids. Oxide-bound (F3): Pb associated with Fe/Mn oxides rose markedly (+30–40%), becoming one of the dominant fractions by day 90. This increase signifies Pb binding to newly formed amorphous Al and Fe hydroxides, with strong evidence of chemisorption and co-precipitation on Al–OH surfaces. These findings align with studies on alum-stabilized mine tailings and sediments (Lei et al., 2022; Wang et al., 2024), where reactive Al phases serve as key Pb sinks. Organic matter-bound (F4): Pb in the organic-bound fraction decreased by 35%–50%, reflecting alum-induced flocculation and precipitation of dissolved organic carbon (DOC). This reduction in DOC limited Pb–organic complexation, redirecting Pb from soluble complexes toward oxide and carbonate associations. Residual/silicate-bound (F5): Minor increases (≤5%) in F5 were observed, indicating some long-term integration of Pb into more recalcitrant matrices, though the short incubation duration limited substantial crystallographic incorporation.

#### 3.4.3 Alum: Al-hydroxide sorption and co-precipitation processes

The fractionation patterns (F1 ↓, F2 ↑, F3 ↑, F4 ↓, slight F5 ↑) indicate that alum immobilized Pb primarily through Al-hydroxide formation, sorption, and DOC flocculation. At T7, rapid hydrolysis of Al^3+^ generated amorphous Al(OH)_3_, creating abundant high-affinity sorption sites that sequestered Pb^2+^ from the exchangeable pool (F1) and increased its association with oxide surfaces (F3).

By T30, continued Al hydrolysis and precipitation strengthened the development of reactive Al- and Fe-oxide surfaces, promoting co-precipitation of Pb with these hydroxides. Simultaneously, alum-induced flocculation of dissolved organic carbon reduced the formation of soluble organo-Pb complexes, contributing to the decline in the organic-bound fraction (F4).

At T90, Pb was predominantly stabilized within the oxide- and carbonate-bound fractions (F2–F3), while slight increases in the residual fraction (F5) suggest gradual incorporation into more recalcitrant mineral phases. These immobilization pathways occurred despite only modest pH changes, consistent with alum-mediated stabilization mechanisms observed in contaminated soils and sediments (Lwin et al., 2023; Lei et al., 2022; Wang et al., 2024; Zhou and Haynes, 2011).

Across the three soil systems, amendment performance followed clear soil-dependent trends. Gypsum promoted rapid Ca-driven stabilization in alkaline San Antonio soils; biochar + lime achieved strong pH-mediated immobilization in acidic Baltimore soils; and alum enhanced sorption and co-precipitation in neutral Detroit soils without major pH alteration. Despite contrasting soil chemistries, all amendments effectively reduced labile Pb (F1) by 40%–60% and increased its retention in carbonate-, oxide-, or organic-bound forms. These findings confirm that tailored amendments leveraging dominant soil geochemical controls, Ca^2+^ activity, pH buffering, and Al-hydroxide formation can achieve sustainable Pb immobilization under diverse urban conditions.

A methodological limitation of this study is the absence of a physical dilution control in which soils were amended with an inert solid at the same mass fraction as the reactive amendments. Consequently, a small contribution of physical dilution to the observed changes in Pb speciation cannot be experimentally excluded. Nevertheless, several lines of evidence suggest that Pb redistribution was driven predominantly by chemical immobilization rather than simple dilution. First, with amendment doses of 3%–5% (w/w), bulk Pb concentrations could at most have been reduced by a few percent, whereas exchangeable Pb (F1) was decreased by 25%–60% and was accompanied by corresponding gains in carbonate-, oxide-, and organic-bound pools (F2–F4). Second, Pb mass balance across all fractions remained stable (92%–103% of bulk Pb) in both control and treated samples, indicating that Pb was internally redistributed rather than lost or diluted from the system. Third, fractionation changes were observed to evolve systematically over time (from 7 to 90 days) and to differ among the three amendments in patterns consistent with their expected geochemical behavior-Ca^2+^-facilitated carbonate formation for gypsum, pH elevation and biochar surface complexation for biochar + lime, and Al-hydroxide sorption and DOC flocculation for alum. Taken together, these observations indicate that reductions in labile Pb and increases in carbonate-, oxide-, and organic-bound fractions primarily reflected amendment-induced chemical stabilization, although future work including an inert mass control would allow any residual physical dilution effects to be more tightly constrained.

### 3.5 Preliminary life cycle analysis (LCA)

The life cycle assessment (LCA) results demonstrate that all amendment-based Pb immobilization treatments have relatively low cradle-to-application environmental impacts, confirming the overall sustainability of the proposed remediation approach. The comparative midpoint impact assessment (Table 2) shows that variations among treatments primarily reflect differences in amendment composition and transport requirements rather than production burdens. Moreover, the traditional alternative, excavation and landfilling of contaminated soil, show larger impacts than the treatment proposed for two (gypsum and alum) of the three amendments used. The biochar-based treatment for the Baltimore soil has impacts that are substantially higher than the landfill scenario in several categories. Although *in situ* immobilization is typically preferred over soil removal from both sustainability and risk-management perspectives, the Baltimore biochar scenario exhibits higher life-cycle impacts than the excavation–and–landfill option for several midpoint categories under the specific conditions evaluated in this study. This outcome is driven almost entirely by long-distance transport of biochar (4,340 km) and the inherently energy-intensive nature of its production. As a result, the biochar scenario shows greater burdens in climate change, fossil resource depletion, particulate matter formation, terrestrial acidification, and freshwater eutrophication relative to the landfill alternative for the for the selected functional unit. However, the landfill scenario remains less favorable for human health and ecosystem damage when full diesel emission profiles are considered, and it does not provide long-term contaminant immobilization benefits. Importantly, the relative performance of the biochar option is highly sensitive to transport distance and amendment loading: local sourcing or lower biochar application rates would substantially reduce its environmental burdens, and in many practical settings would shift the comparison in favor of *in situ* stabilization. These results emphasize that the environmental competitiveness of biochar-based treatments depends strongly on supply-chain configuration rather than intrinsic disadvantages of the amendment itself.

**TABLE 2:** Comparative results of ReCiPe 2016 midpoint impact assessment per functional unit and for the excavation-landfilling option.

Impact Category	Unit	Detroit – Alum	San Antonio – Gypsum	Baltimore – Biochar + Ca(OH)_2_	Landfill (450 kg soil, 80 km)
Climate change (GWP)	kg CO_2_-eq	0.05–0.07	1.4–1.6	9.5–11	2.5–3.1
Human health	DALY	(0.7–1.1)×10^–8^	(2.1–2.4)×10^–7^	(1.4–1.7)×10^–6^	(3.0–4.5)×10^–7^
Ecosystem quality	species·yr	(2.0–2.8)×10^–9^	(5.6–6.4)×10^–8^	(3.8–4.4)×10^–7^	(6.0–9.0)×10^–8^
PM_2_._5_ formation	kg PM_2_._5_-eq	(3–5)×10^–7^	(1.0–1.3)×10^–5^	(1.0–1.3)×10^–4^	(5.0–7.0)×10^–5^
Terrestrial acidification	kg SO_2_-eq	2.6 × 10^−4^	8.9 × 10^−3^	8.9 × 10^−2^	3.0–4.5 × 10^−3^
Freshwater eutrophication	kg P-eq	(3–5)×10^–6^	(2.5–3.6)×10^–5^	(1–1.4)×10^–5^	(1.5–2.0)×10^–4^
Fossil resource use	MJ surplus	0.8–1.2	8–12	1,050–1,300	35–45

Another observation from Table 2 is that transportation contributed the largest share of total impacts, particularly to global warming potential and resource depletion, due to the energy intensity of long-distance shipping and this effect was most pronounced for biochar-based treatments, where transport distances from production facilities significantly influenced overall emissions.

By contrast, amendment production contributed minimally to total impacts, indicating that the materials themselves, pistachio-shell biochar + lime, alum, and gypsum, are inherently low-impact. When normalized to the treatment of 1 m^2^ of contaminated soil, the total greenhouse gas emissions remained small, even under a hypothetical annual re-application scenario.

Overall, these results highlight that the amendment selection strategy, especially if amendments are sourced locally, not only enhances Pb stabilization but also aligns with sustainable remediation goals by maintaining low environmental footprints across all tested soil–amendment systems.

## 4 Conclusion

This study demonstrated that amendment selection tailored to site-specific soil geochemistry can effectively and sustainably immobilize Pb in contaminated urban soils. Across the three contrasting soil systems, alkaline San Antonio, acidic Baltimore, and neutral Detroit, distinct amendments (gypsum, biochar + lime, and alum, respectively) consistently transformed Pb from labile, exchangeable forms into more stable carbonate-, oxide-, and organic-bound fractions. Despite their differing geochemical environments, all treatments achieved significant (∼50%, range 40%–60%) reductions in bioavailable Pb within 90 days, confirming the robustness of targeted *in situ* immobilization strategies.

These reductions in the exchangeable (F1) fraction have direct implications for human health risk. The F1 pool is the strongest predictor of both *in-vitro* bioaccessibility and *in-vivo* relative bioavailability, and multiple recent studies demonstrate that 40%–60% decreases in F1-Pb correspond to substantial declines in bioaccessible Pb under gastric conditions (Goncalves et al., 2023; Lake et al., 2021; Li et al., 2020). Such reductions are also consistent with lower absorbed dose estimates produced by the IEUBK model (U.S. EPA, 2007; Drexler and Brattin, 2007). Therefore, the fractionation shifts observed in this study, namely, decreases in exchangeable Pb accompanied by increases in carbonate- and oxide-bound pools, provide strong evidence that the tested amendments not only immobilize Pb geochemically but also meaningfully reduce its bioavailability and associated human exposure risk.

Mechanistic interpretation revealed that immobilization proceeded through complementary pathways: Ca^2+^-driven carbonate precipitation and co-precipitation with Fe/Mn oxides in San Antonio; pH-mediated carbonate formation and surface complexation with biochar functional groups in Baltimore; and Al-hydroxide surface sorption and organic carbon flocculation in Detroit. These processes collectively minimized Pb mobility without inducing secondary soil degradation or pH instability.

A preliminary life cycle assessment further showed that amendment-based immobilization has a substantially lower environmental footprint than excavation-and-landfilling for most scenarios, with transportation distance emerging as the dominant contributor to impacts. These results reinforce that locally sourced amendments can deliver both geochemical effectiveness and sustainability benefits.

By integrating sequential extraction, pH evolution, and statistical analyses, this study establishes a mechanistic linkage between amendment chemistry and soil-specific controls on Pb immobilization. The results demonstrate that targeted amendments can reduce labile Pb by 40%–60% through geochemical pathways consistent with soil mineralogy and pH buffering capacity. This approach offers a scalable, low-cost, and environmentally compatible strategy for mitigating Pb exposure in urban environments. The findings can inform soil management and policy frameworks focused on sustainable remediation of legacy-contaminated urban areas. Future work should extend these findings through long-term field trials and advanced spectroscopic analyses to confirm mineralogical transformations and assess amendment durability under natural weathering conditions.

## Data Availability

The original contributions presented in the study are included in the article/Supplementary Material, further inquiries can be directed to the corresponding author.
