# Supplementary material for: Geochemical fate of lead in contaminated residential soils following application of amendments for lead immobilization
Source: Front Chem. 2026 Feb 5;14:1742013. doi: 10.3389/fchem.2026.1742013 (PMC12916109; doi:10.3389/fchem.2026.1742013)
Supplement: Supplementary file 1 [file Table1.docx]

Supplementary Material

**Geochemical fate of lead in contaminated residential soils following application of amendments for lead immobilization**

Hadeer Saleh^1^, Washington Braida^1^, Zhiming Zhang^2^, Rupali Datta^3^, and Dibyendu Sarkar^1*^

^1^Department of Civil, Environmental, and Ocean Engineering, Stevens Institute of Technology,

Hoboken, NJ, USA

^2^Department of Civil and Environmental Engineering, Rowan University, Glassboro, NJ 08028, USA

^3^Department of Biological Sciences, Michigan Technological University, Houghton, MI, USA

* **Correspondence:**
Dibyendu Sarkar
[dsarkar@stevens.edu](mailto:dsarkar@stevens.edu)

**Table S1.** Physicochemical properties of Pb-contaminated soils from San Antonio, TX. Values are mean ± standard deviation (n=3).

| **Sample** | **Clay (%)** | **pH** | **EC (µS cm⁻¹)** | **CEC**  **(meq 100 g⁻¹)** | **SOM (%)** | **Pb**  **(mg kg⁻¹)** | **Al**  **(mg kg⁻¹)** | **Fe**  **(mg kg⁻¹)** | **P**  **(mg kg⁻¹)** | **Ca**  **(mg kg⁻¹)** | **Mg**  **(mg kg⁻¹)** |
| --- | --- | --- | --- | --- | --- | --- | --- | --- | --- | --- | --- |
| SA1 | 7.3 ± 0.5 | 7.70 ± 0.20 | 1050 ± 14 | 13.3 ± 1.8 | 7.7 ± 0.4 | 2030 ± 110 | 22 800 ± 1000 | 10 700 ± 1700 | 370 ± 35 | 22 200 ± 1600 | 1950 ± 710 |
| SA2 | 14.4 ± 1.1 | 7.80 ± 0.01 | 770 ± 19 | 12.2 ± 0.9 | 9.9 ± 0.4 | 6780 ± 80 | 20 500 ± 970 | 9870 ± 1700 | 290 ± 40 | 28 200 ± 2100 | 1290 ± 270 |
| SA3 | 15.0 ± 1.1 | 7.60 ± 0.01 | 610 ± 5 | 12.0 ± 1.6 | 8.8 ± 0.6 | 5690 ± 90 | 17 300 ± 2300 | 9440 ± 520 | 470 ± 30 | 21 200 ± 1400 | 700 ± 40 |
| SA4 | 8.1 ± 0.6 | 7.50 ± 0.10 | 340 ± 20 | 12.2 ± 0.8 | 5.2 ± 0.3 | 360 ± 40 | 25 300 ± 1600 | 4340 ± 600 | 420 ± 20 | 54 300 ± 2300 | 1330 ± 80 |
| SA5 | 7.1 ± 0.5 | 7.40 ± 0.01 | 420 ± 30 | 17.6 ± 0.6 | 10.2 ± 0.4 | 840 ± 40 | 25 000 ± 960 | 12 500 ± 1100 | 480 ± 20 | 49 900 ± 2100 | 970 ± 110 |
| SA6 | 8.7 ± 0.6 | 7.50 ± 0.10 | 330 ± 10 | 22.5 ± 1.5 | 12.9 ± 0.4 | 2660 ± 340 | 18 800 ± 1700 | 7930 ± 520 | 580 ± 10 | 33 600 ± 2700 | 1000 ± 140 |
| SA7 | 22.5 ± 1.6 | 7.60 ± 0.10 | 300 ± 40 | 26.0 ± 2.3 | 5.3 ± 0.4 | 7770 ± 510 | 23 600 ± 1200 | 8180 ± 370 | 580 ± 30 | 72 100 ± 1400 | 1130 ± 140 |
| SA8 | 65.2 ± 4.9 | 7.90 ± 0.01 | 230 ± 20 | 41.7 ± 1.3 | 6.8 ± 0.2 | 420 ± 30 | 23 600 ± 1500 | 6910 ± 380 | 280 ± 10 | 62 100 ± 1900 | 1630 ± 330 |
| SA9 | 16.7 ± 1.2 | 7.80 ± 0.10 | 220 ± 10 | 15.4 ± 0.4 | 6.4 ± 0.2 | 470 ± 40 | 25 600 ± 1000 | 18 800 ± 1200 | 390 ± 20 | 53 800 ± 1700 | 1490 ± 190 |
| SA10 | 14.6 ± 1.1 | 7.50 ± 0.10 | 320 ± 30 | 14.9 ± 0.1 | 11.0 ± 0.2 | 7680 ± 580 | 19 700 ± 760 | 3020 ± 180 | 440 ± 20 | 20 700 ± 1900 | 1540 ± 300 |

**Table S2.** Physicochemical properties of Pb-contaminated soils from Baltimore, MD. Values are mean ± standard deviation (n=3).

| **Sample** | **Clay (%)** | **pH** | **EC (µS/cm)** | **CEC (meq/100 g)** | **SOM (%)** | **Pb (mg/kg)** | **Al (mg/kg)** | **Fe (mg/kg)** | **P (mg/kg)** | **Ca (mg/kg)** | **Mg (mg/kg)** |
| --- | --- | --- | --- | --- | --- | --- | --- | --- | --- | --- | --- |
| B1 | 4.9 ± 0.4 | 6.4 ± 0.1 | 660 ± 30 | 6.5 ± 0.4 | 2.3 ± 0.6 | 320 ± 40 | 30 800 ± 4000 | 19 300 ± 2600 | 3770 ± 510 | 11 800 ± 1500 | 1650 ± 330 |
| B2 | 5.2 ± 0.4 | 5.2 ± 0.1 | 160 ± 9 | 5.7 ± 0.4 | 2.5 ± 0.7 | 39 ± 4 | 22 100 ± 2900 | 14 000 ± 1800 | 2460 ± 320 | 4770 ± 640 | 1210 ± 220 |
| B3 | 3.4 ± 0.3 | 6.1 ± 0.0 | 260 ± 10 | 8.3 ± 0.7 | 1.9 ± 0.5 | 2560 ± 290 | 24 300 ± 3200 | 20 700 ± 2700 | 3300 ± 440 | 13 400 ± 1800 | 2430 ± 460 |
| B4 | 2.1 ± 0.1 | 6.3 ± 0.0 | 110 ± 2 | 6.2 ± 0.9 | 0.6 ± 0.1 | 590 ± 70 | 23 000 ± 3200 | 18 600 ± 2500 | 8620 ± 1150 | 7340 ± 910 | 1170 ± 260 |
| B5 | 2.2 ± 0.2 | 6.2 ± 0.1 | 280 ± 20 | 9.6 ± 0.5 | 1.2 ± 0.4 | 2790 ± 300 | 28 000 ± 3700 | 15 700 ± 2100 | 3330 ± 440 | 26 100 ± 3300 | 2240 ± 410 |
| B6 | 2.9 ± 0.3 | 6.2 ± 0.1 | 240 ± 20 | 7.3 ± 0.5 | 1.6 ± 0.4 | 680 ± 80 | 28 300 ± 3800 | 17 300 ± 2200 | 6850 ± 910 | 6400 ± 850 | 1450 ± 300 |
| B7 | 4.3 ± 0.3 | 6.2 ± 0.1 | 100 ± 6 | 6.4 ± 0.5 | 1.9 ± 0.5 | 2130 ± 220 | 30 000 ± 4000 | 17 000 ± 2300 | 3240 ± 430 | 5510 ± 730 | 1610 ± 310 |
| B8 | 4.2 ± 0.4 | 6.2 ± 0.1 | 720 ± 50 | 8.0 ± 0.6 | 1.8 ± 0.6 | 390 ± 40 | 22 400 ± 3000 | 20 800 ± 2700 | 9190 ± 1220 | 8920 ± 1180 | 1570 ± 340 |
| B9 | 3.8 ± 0.3 | 5.9 ± 0.1 | 130 ± 8 | 5.1 ± 0.4 | 1.8 ± 0.5 | 5370 ± 660 | 22 500 ± 2900 | 15 300 ± 2000 | 7840 ± 1150 | 9370 ± 1250 | 1570 ± 330 |
| B10 | 2.7 ± 0.2 | 5.4 ± 0.1 | 260 ± 20 | 6.9 ± 0.5 | 1.8 ± 0.5 | 3960 ± 490 | 21 700 ± 2800 | 20 000 ± 2400 | 5800 ± 660 | 5640 ± 700 | 1580 ± 360 |

| **Sample** | **Clay (%)** | **pH** | **EC (µS/cm)** | **CEC (meq/100 g)** | **SOM (%)** | **Pb (mg/kg)** | **Al (mg/kg)** | **Fe (mg/kg)** | **P (mg/kg)** | **Ca (mg/kg)** | **Mg (mg/kg)** |
| --- | --- | --- | --- | --- | --- | --- | --- | --- | --- | --- | --- |
| D1 | 16.5 ± 1.4 | 7.7 ± 0.0 | 340 ± 10 | 47.0 ± 1.9 | 7.6 ± 0.3 | 365 ± 22 | 36 900 ± 1200 | 14 600 ± 500 | 1220 ± 40 | 17 500 ± 800 | 2120 ± 70 |
| D2 | 14.3 ± 1.2 | 7.8 ± 0.1 | 360 ± 20 | 32.8 ± 1.2 | 4.7 ± 0.5 | 690 ± 45 | 37 500 ± 1200 | 10 200 ± 350 | 740 ± 30 | 14 900 ± 600 | 1640 ± 70 |
| D3 | 19.6 ± 1.6 | 7.9 ± 0.0 | 490 ± 20 | 53.8 ± 1.4 | 10.4 ± 0.2 | 590 ± 35 | 37 200 ± 1300 | 4450 ± 150 | 320 ± 10 | 10 700 ± 770 | 630 ± 30 |
| D4 | 9.1 ± 0.8 | 7.7 ± 0.1 | 510 ± 20 | 43.6 ± 2.1 | 9.8 ± 0.5 | 124 ± 8 | 38 200 ± 1400 | 15 600 ± 540 | 1070 ± 70 | 13 100 ± 750 | 2040 ± 100 |
| D5 | 10.9 ± 0.9 | 7.2 ± 0.1 | 300 ± 10 | 40.8 ± 1.6 | 8.4 ± 0.3 | 1570 ± 170 | 36 300 ± 2100 | 4460 ± 260 | 350 ± 20 | 9360 ± 520 | 1120 ± 70 |
| D6 | 7.3 ± 0.6 | 7.3 ± 0.1 | 340 ± 10 | 11.6 ± 0.8 | 7.1 ± 0.6 | 1100 ± 96 | 35 000 ± 2200 | 8580 ± 370 | 710 ± 30 | 12 300 ± 620 | 1400 ± 90 |
| D7 | 20.0 ± 1.6 | 7.6 ± 0.1 | 840 ± 30 | 64.3 ± 3.2 | 10.3 ± 0.4 | 660 ± 82 | 34 700 ± 1200 | 12 100 ± 520 | 840 ± 30 | 13 400 ± 560 | 1870 ± 80 |
| D8 | 14.2 ± 1.2 | 7.1 ± 0.1 | 230 ± 10 | 30.0 ± 1.8 | 8.0 ± 0.2 | 430 ± 58 | 34 600 ± 2400 | 9350 ± 520 | 1420 ± 70 | 13 400 ± 960 | 1470 ± 110 |
| D9 | 6.8 ± 0.6 | 7.9 ± 0.0 | 780 ± 10 | 15.5 ± 1.1 | 6.0 ± 0.2 | 400 ± 46 | 36 100 ± 3100 | 9050 ± 410 | 820 ± 30 | 18 700 ± 850 | 1720 ± 100 |
| D10 | 16.4 ± 1.4 | 6.3 ± 0.1 | 580 ± 10 | 38.2 ± 2.2 | 7.6 ± 0.4 | 1130 ± 140 | 33 600 ± 1100 | 8060 ± 280 | 790 ± 30 | 8830 ± 300 | 980 ± 60 |

**Table S3.** Physicochemical properties of Pb-contaminated soils from Detroit, MI. Values are mean ± standard deviation (n=3).

| **Sample ID** | **Control (T7)** | **Treated (T7)** | **Control (T30)** | **Treated (T30)** | **Control (T90)** | **Treated (T90)** |
| --- | --- | --- | --- | --- | --- | --- |
| SA1 | 7.78 ± 0.11 | 7.89 ± 0.34 | 7.72 ± 0.41 | 7.79 ± 0.15 | 7.72 ± 0.41 | 7.75 ± 0.05 |
| SA2 | 7.62 ± 0.20 | 7.65 ± 0.25 | 7.64 ± 0.23 | 7.83 ± 0.33 | 7.64 ± 0.23 | 7.71 ± 0.12 |
| SA3 | 7.37 ± 0.43 | 7.48 ± 0.77 | 7.43 ± 0.14 | 7.62 ± 0.29 | 7.43 ± 0.14 | 7.58 ± 0.13 |
| SA4 | 7.75 ± 0.62 | 7.79 ± 0.42 | 7.84 ± 0.27 | 7.86 ± 0.24 | 7.84 ± 0.27 | 7.82 ± 0.08 |
| SA5 | 7.57 ± 0.65 | 7.64 ± 0.98 | 7.59 ± 0.42 | 7.64 ± 0.36 | 7.59 ± 0.42 | 7.67 ± 0.14 |
| SA6 | 7.48 ± 0.53 | 7.58 ± 0.09 | 7.49 ± 0.29 | 7.71 ± 0.14 | 7.49 ± 0.29 | 7.62 ± 0.09 |
| SA7 | 7.72 ± 0.17 | 7.81 ± 0.14 | 7.68 ± 0.18 | 7.91 ± 0.18 | 7.68 ± 0.18 | 7.84 ± 0.12 |
| SA8 | 7.74 ± 0.51 | 7.98 ± 0.58 | 7.69 ± 0.33 | 7.93 ± 0.39 | 7.69 ± 0.33 | 7.83 ± 0.14 |
| SA9 | 7.63 ± 0.24 | 7.81 ± 0.97 | 7.60 ± 0.14 | 7.76 ± 0.17 | 7.60 ± 0.14 | 7.71 ± 0.10 |
| SA10 | 7.58 ± 0.40 | 7.69 ± 0.43 | 7.62 ± 0.22 | 7.78 ± 0.34 | 7.62 ± 0.22 | 7.76 ± 0.19 |

**Table S4.** Evolution of soil pH in San Antonio soils during amendment incubation. Values are mean ± standard deviation (n = 3).

**Table S5.** Evolution of soil pH in Baltimore soils during amendment incubation. Values are mean ± standard deviation (n = 3).

| **Sample ID** | **Control (T7)** | **Treated (T7)** | **Control (T30)** | **Treated (T30)** | **Control (T90)** | **Treated (T90)** |
| --- | --- | --- | --- | --- | --- | --- |
| B1 | 6.61 ± 0.03 | 7.09 ± 0.08 | 6.90±0.40 | 7.12±0.60 | 6.83±0.11 | 7.14±0.25 |
| B2 | 5.88 ± 0.04 | 6.80 ± 0.04 | 6.23±0.50 | 7.01±0.40 | 6.77±0.13 | 7.00±0.21 |
| B3 | 6.31 ± 0.06 | 7.38 ± 0.07 | 6.72±0.30 | 7.29±0.50 | 6.85±0.12 | 7.28±0.16 |
| B4 | 6.68 ± 0.07 | 7.13 ± 0.09 | 7.02±0.60 | 7.33±0.70 | 7.06±0.21 | 7.35±0.23 |
| B5 | 6.13 ± 0.02 | 6.88 ± 0.02 | 6.54±0.80 | 7.00±0.30 | 6.67±0.18 | 7.02±0.31 |
| B6 | 6.35 ± 0.07 | 7.09 ± 0.12 | 6.76±0.60 | 7.11±0.20 | 6.88±0.14 | 7.10±0.24 |
| B7 | 6.74 ± 0.20 | 7.12 ± 0.08 | 6.97±0.50 | 7.19±0.60 | 7.01±0.19 | 7.21±0.12 |
| B8 | 6.34 ± 0.03 | 7.01 ± 0.05 | 6.55±0.40 | 7.03±0.50 | 6.78±0.17 | 7.02±0.16 |
| B9 | 6.41 ± 0.06 | 6.92 ± 0.03 | 6.48±0.20 | 6.90±0.40 | 6.81±0.11 | 6.92±0.14 |
| B10 | 5.73 ± 0.08 | 6.49 ± 0.09 | 6.25±0.30 | 6.71±0.40 | 6.49±0.10 | 6.70±0.13 |

**Table S6.** Evolution of soil pH in Detroit soils during amendment incubation. Values are mean ± standard deviation (n = 3).

| **Sample ID** | **Control (T7)** | **Treated (T7)** | **Control (T30)** | **Treated (T30)** | **Control (T90)** | **Treated (T90)** |
| --- | --- | --- | --- | --- | --- | --- |
| D1 | 6.70±0.03 | 7.01±0.02 | 7.40±0.21 | 7.47±0.31 | 7.72 ± 0.41 | 7.75 ± 0.05 |
| D2 | 6.54±0.08 | 6.77±0.04 | 7.21±0.32 | 7.19±0.14 | 7.64 ± 0.23 | 7.71 ± 0.12 |
| D3 | 6.40±0.09 | 6.42±0.09 | 7.49±0.11 | 7.44±0.17 | 7.43 ± 0.14 | 7.58 ± 0.13 |
| D4 | 6.61±0.11 | 6.92±0.05 | 7.10±0.09 | 6.98±0.11 | 7.84 ± 0.27 | 7.82 ± 0.08 |
| D5 | 6.15±0.02 | 6.30±0.07 | 7.02±0.12 | 6.93±0.09 | 7.59 ± 0.42 | 7.67 ± 0.14 |
| D6 | 6.18±0.04 | 6.65±0.12 | 7.20±0.08 | 7.17±0.12 | 7.49 ± 0.29 | 7.62 ± 0.09 |
| D7 | 6.48±0.05 | 6.88±0.10 | 7.41±0.14 | 7.37±0.11 | 7.68 ± 0.18 | 7.84 ± 0.12 |
| D8 | 6.36±0.08 | 6.49±0.08 | 6.72±0.20 | 6.67±0.18 | 7.69 ± 0.33 | 7.83 ± 0.14 |
| D9 | 6.52±0.07 | 6.81±0.09 | 7.31±0.13 | 7.37±0.21 | 7.60 ± 0.14 | 7.71 ± 0.10 |
| D10 | 6.09±0.06 | 6.10±0.04 | 6.42±0.24 | 6.33±0.07 | 7.62 ± 0.22 | 7.76 ± 0.19 |

| **Sample ID** | **F1 – Soluble + Exchangeable** | | **F2 – Carbonate-bound** | | **F3 – Oxide-bound** | | **F4 – Organic Matter** | | **F5 – Silicate-bound** | |
| --- | --- | --- | --- | --- | --- | --- | --- | --- | --- | --- |
|  | Control | Incubated | Control | Incubated | Control | Incubated | Control | Incubated | Control | Incubated |
| SA1 | 205 ± 8.4 | 167 ± 10 | 483 ± 23 | 497 ± 29 | 636 ± 24 | 814 ± 34 | 505 ± 22 | 269 ± 11 | 248 ± 6 | 268± 12 |
| SA2 | 599 ± 25 | 480 ± 28 | 1811 ± 87 | 1901 ± 108 | 1132 ± 52 | 2312 ± 60 | 1944 ± 76 | 848.5 ± 40 | 1241 ± 50 | 1202 ± 53 |
| SA3 | 488 ± 20 | 298 ± 18 | 1204 ± 57 | 1291 ± 74 | 1320 ± 25 | 1388 ± 59 | 1555 ± 62 | 1305 ± 55 | 1031 ± 42 | 1312 ± 59 |
| SA4 | 24 ± 1 | 17 ± 1 | 132 ± 6 | 124 ± 7 | 122 ± 4 | 144 ± 7 | 39 ± 3 | 21.0 ± 0.9 | 41 ± 1 | 54± 4 |
| SA5 | 73 ± 3 | 58 ± 3 | 251 ± 12 | 276 ± 15 | 147 ± 5 | 184 ± 9 | 282 ± 12 | 204 ± 10 | 81 ± 4 | 97± 7 |
| SA6 | 301 ± 12 | 224 ± 13 | 841 ± 38 | 871 ± 50 | 212 ± 8 | 297 ± 15 | 973 ± 38 | 847 ± 38 | 321 ± 19 | 403± 17 |
| SA7 | 612 ± 25 | 478 ± 28 | 3086 ± 154 | 2940 ± 168 | 1503 ± 58 | 1961± 85 | 1009 ± 40 | 878 ± 37 | 1548 ± 67 | 1432 ± 87 |
| SA8 | 28 ± 1 | 22 ± 1 | 138 ± 7 | 156 ± 8 | 8.0 ± 0.3 | 22.0 ± 0.9 | 73 ± 3 | 45 ± 2 | 166 ± 7 | 171 ± 7 |
| SA9 | 49 ± 2 | 37 ± 2 | 211 ± 10 | 204 ± 11 | 26 ± 2 | 51 ± 3 | 91 ± 4 | 77 ± 3 | 92 ± 4 | 101 ± 5 |
| SA10 | 854 ± 35 | 647 ± 38 | 2474 ± 120 | 2348 ± 133 | 614 ± 24 | 523 ± 22 | 2579 ± 148 | 2116 ± 95 | 1814 ± 52 | 2001± 85 |

**Table S7**. Geochemical fractionation of Pb in San Antonio soils after 7 days of incubation (mg/kg). Values are mean ± SD (n = 3).

**Table S8**. Geochemical fractionation of Pb in San Antonio soils after 30 days of incubation (mg/kg). Values are mean ± SD (n = 3).

| **Sample ID** | **F1 – Soluble + Exchangeable** | | **F2 – Carbonate-bound** | | **F3 – Oxide-bound** | | **F4 – Organic Matter** | | **F5 – Silicate-bound** | |
| --- | --- | --- | --- | --- | --- | --- | --- | --- | --- | --- |
|  | Control | Incubated | Control | Incubated | Control | Incubated | Control | Incubated | Control | Incubated |
| SA1 | 213± 15 | 163± 10 | 468± 33 | 509± 40 | 621± 44 | 842 ± 61 | 487± 35 | 261 ± 18 | 173± 12 | 201 ± 15 |
| SA2 | 622± 44 | 477± 32 | 1799 ± 131 | 1917± 96 | 1147± 80 | 2389 ± 172 | 1958± 143 | 814 ± 57 | 1295± 93 | 1253 ± 90 |
| SA3 | 503 ± 35 | 284 ± 19 | 1233 ± 84 | 1345 ± 20 | 1326 ± 95 | 1420 ± 103 | 1524± 113 | 1211 ± 87 | 1065 ± 77 | 1201 ± 86 |
| SA4 | 26± 1 | 18 ± 1 | 129 ± 9 | 137± 11 | 126 ± 8 | 152 ± 10 | 41± 2 | 18 ± 1 | 43± 3 | 49 ± 3 |
| SA5 | 71 ± 5 | 52 ± 3 | 258± 18 | 283± 20 | 151 ± 11 | 189 ± 13 | 294± 19 | 198± 14 | 89± 6 | 90 ± 6 |
| SA6 | 295± 22 | 217± 14 | 873 ± 60 | 891 ± 64 | 208± 14 | 312 ± 22 | 1002± 72 | 810 ± 58 | 350± 25 | 374 ± 26 |
| SA7 | 601± 43 | 482 ± 29 | 3101 ± 223 | 3022 ± 217 | 1489 ± 107 | 2009 ± 144 | 987± 71 | 801 ± 58 | 1601 ± 111 | 1579 ± 114 |
| SA8 | 31± 2 | 18 ± 1 | 142 ± 10 | 161± 11 | 10.0± 0.6 | 26 ± 1 | 68 ± 5 | 37± 2 | 175± 11 | 179± 13 |
| SA9 | 47± 4 | 32± 2 | 200 ± 14 | 210± 14 | 22 ± 1 | 54 ± 3 | 97± 7 | 71 ± 5 | 101 ± 7 | 106 ± 7 |
| SA10 | 827± 55 | 611± 40 | 2463 ± 177 | 2499 ± 180 | 469 ± 35 | 547 ± 41 | 2518± 181 | 2013 ± 145 | 1375 ± 101 | 1678 ± 119 |

**Table S9**. Geochemical fractionation of Pb in San Antonio soils after 90 days of incubation (mg/kg). Values are mean ± SD (n = 3).

| **Sample ID** | **F1 – Soluble + Exchangeable** | | **F2 – Carbonate-bound** | | **F3 – Oxide-bound** | | **F4 – Organic Matter** | | **F5 – Silicate-bound** | |
| --- | --- | --- | --- | --- | --- | --- | --- | --- | --- | --- |
|  | Control | Incubated | Control | Incubated | Control | Incubated | Control | Incubated | Control | Incubated |
| SA1 | 217± 15 | 163 ± 10 | 457± 22 | 509± 40 | 618± 14 | 842± 61 | 472± 34 | 261 ± 18 | 166± 7 | 201± 15 |
| SA2 | 617± 19 | 477± 32 | 1732± 60 | 1917± 96 | 1157± 12 | 2389 ± 172 | 1965± 22 | 814 ± 57 | 1261± 9 | 1253 ± 90 |
| SA3 | 486±24 | 284 ± 19 | 1274± 31 | 1345 ± 20 | 1339± 92 | 1420 ± 103 | 1500± 84 | 1211 ± 87 | 1024± 13 | 1201 ± 86 |
| SA4 | 28± 5 | 18 ± 1 | 131± 14 | 137± 11 | 137±5 | 152 ± 10 | 45± 3 | 18 ± 1 | 48± 2 | 49 ± 3 |
| SA5 | 73± 4 | 52 ± 3 | 251± 11 | 283± 20 | 145± 4 | 189 ± 13 | 300± 13 | 198 ± 14 | 93± 6 | 90 ± 6 |
| SA6 | 303± 15 | 217 ± 14 | 862± 18 | 891 ± 64 | 203± 17 | 312 ± 22 | 1013± 61 | 810 ± 58 | 341± 21 | 374 ± 26 |
| SA7 | 589± 45 | 482 ± 29 | 3111± 84 | 3022 ± 217 | 1569±22 | 2009 ± 144 | 993± 15 | 801 ± 58 | 1635± 47 | 1579 ± 114 |
| SA8 | 32± 1 | 18 ± 1 | 149± 16 | 161± 11 | 10.0± 0.2 | 26 ± 2 | 72± 4 | 37 ± 2 | 161± 3 | 179± 13 |
| SA9 | 52± 2 | 32 ± 2 | 194± 6 | 210± 14 | 18± 1 | 54 ± 4 | 100± 5 | 71 ± 5 | 97± 6 | 106 ± 8 |
| SA10 | 810± 65 | 611 ± 40 | 2474± 30 | 2499 ± 180 | 451± 11 | 547± 41 | 2542± 98 | 2013 ± 145 | 1362± 22 | 1678 ± 119 |

**Table S10**. Geochemical fractionation of Pb in Baltimore soils after 7 days of incubation (mg/kg). Values are mean ± SD (n = 3).

| **Sample ID** | **F1 – Soluble + Exchangeable** | | **F2 – Carbonate-bound** | | **F3 – Oxide-bound** | | **F4 – Organic Matter** | | **F5 – Silicate-bound** | |
| --- | --- | --- | --- | --- | --- | --- | --- | --- | --- | --- |
|  | Control | Incubated | Control | Incubated | Control | Incubated | Control | Incubated | Control | Incubated |
| B1 | 101 ± 12 | 79 ± 6 | 44 ± 2 | 74± 5 | 59± 2 | 42 ± 2 | 68 ± 4 | 78 ± 1 | 20 ± 1 | 19.4 ± 0.7 |
| B2 | 19.1 ± 0.8 | 13 ± 1 | 4.35± 0.05 | 7.11± 0.62 | 3.18 ± 0.92 | 2.17 ± 0.54 | 8.01 ± 0.32 | 11 ± 1 | 5.15 ± 0.44 | 6.14 ± 0.34 |
| B3 | 1187± 61 | 862± 61 | 338± 11 | 389± 29 | 571 ± 23 | 533 ± 48 | 362 ± 14 | 413 ± 38 | 126 ± 8 | 128 ± 6 |
| B4 | 187± 9 | 144± 11 | 39 ± 2 | 55.6 ± 0.4 | 302 ± 17 | 288 ± 15 | 25.2± 1.3 | 33.5 ± 0.2 | 10 ± 1 | 12 .0± 0.1 |
| B5 | 110± 29 | 889± 51 | 576 ± 32 | 598 ± 45 | 833 ± 61 | 770 ± 65 | 183 ± 63 | 194 ± 12 | 70 ± 3 | 73 ± 3 |
| B6 | 249 ± 12 | 194± 14 | 93 ± 2 | 133 ± 10 | 217 ± 10 | 196 ± 7 | 64 ± 2 | 60 ± 2 | 27± 1 | 29.3 ± 0.2 |
| B7 | 828 ± 21 | 671± 51 | 486± 18 | 545 ± 41 | 363 ± 12 | 344 ± 33 | 312 ± 24 | 325 ± 26 | 180 ± 4 | 182 ± 12 |
| B8 | 156 ± 3 | 107 ± 8 | 105 ± 2 | 126 ± 2 | 59± 4 | 45 ± 1 | 37 ± 2 | 48± 2 | 29 ± 2 | 27.9± 0.6 |
| B9 | 2553 ± 72 | 2220± 74 | 641 ± 37 | 773 ± 66 | 1257 ± 79 | 1007 ± 43 | 523 ± 21 | 544± 44 | 331 ± 16 | 333 ± 20 |
| B10 | 1934± 24 | 1580± 25 | 187± 15 | 213 ± 10 | 1263 ± 70 | 1230 ± 100 | 400 ± 23 | 417 ± 31 | 155± 3 | 157 ± 42 |

**Table S11**. Geochemical fractionation of Pb in Baltimore soils after 30 days of incubation (mg/kg). Values are mean ± SD (n = 3).

| **Sample ID** | **F1 – Soluble + Exchangeable** | | **F2 – Carbonate-bound** | | **F3 – Oxide-bound** | | **F4 – Organic Matter** | | **F5 – Silicate-bound** | |
| --- | --- | --- | --- | --- | --- | --- | --- | --- | --- | --- |
|  | Control | Incubated | Control | Incubated | Control | Incubated | Control | Incubated | Control | Incubated |
| B1 | 100 ± 7 | 64± 4 | 41± 3 | 68± 5 | 54 ± 4 | 38± 2 | 75 ± 5 | 84± 7 | 22.1 ± 1.7 | 19± 2 |
| B2 | 17± 1 | 11± 0.92 | 5.24± 0.29 | 8.2 ± 0.9 | 4.05 ± 0.23 | 1.78 ± 0.14 | 5.32 ± 0.48 | 9.16 ± 0.69 | 2.93 ± 0.24 | 3.45 ± 0.26 |
| B3 | 1158 ± 96 | 729 ± 55 | 330± 23 | 413 ± 30 | 575 ± 43 | 498 ± 18 | 369 ± 27 | 428 ± 31 | 125± 9 | 127 ± 8 |
| B4 | 179± 12 | 122± 9 | 47± 3 | 64 ± 5 | 306 ± 21 | 253± 15 | 19 ± 2 | 38 ± 2 | 8.84 ± 0.66 | 11.1± 0.8 |
| B5 | 1129 ± 84 | 753± 56 | 569± 45 | 631 ± 42 | 788± 59 | 714± 61 | 187 ± 14 | 203 ± 16 | 66± 3 | 68 ± 5 |
| B6 | 250± 18 | 169± 13 | 94± 7 | 161± 11 | 221± 16 | 180± 13 | 58 ± 4 | 69 ± 5 | 26 ± 1 | 24± 1.5 |
| B7 | 817± 62 | 577± 43 | 476± 35 | 610 ± 45 | 367 ± 26 | 316± 22 | 306± 21 | 373 ± 28 | 184 ± 12 | 182 ± 13 |
| B8 | 177± 12 | 94± 7 | 102± 6 | 153 ± 11 | 54± 4 | 41± 3 | 31± 2 | 56 ± 4 | 24 ± 2 | 24 ± 2 |
| B9 | 2604± 195 | 1896± 141 | 624± 46 | 870 ± 65 | 1261± 90 | 912± 62 | 516± 38 | 600 ± 42 | 330 ± 25 | 333 ± 23 |
| B10 | 1903±142 | 1395±101 | 190± 14 | 253 ± 16 | 1257± 87 | 1225± 91 | 407± 30 | 483 ± 31 | 159 ± 12 | 157 ± 12 |

**Table S12**. Geochemical fractionation of Pb in Baltimore soils after 90 days of incubation (mg/kg). Values are mean ± SD (n = 3).

| **Sample ID** | **F1 – Soluble + Exchangeable** | | **F2 – Carbonate-bound** | | **F3 – Oxide-bound** | | **F4 – Organic Matter** | | **F5 – Silicate-bound** | |
| --- | --- | --- | --- | --- | --- | --- | --- | --- | --- | --- |
|  | Control | Incubated | Control | Incubated | Control | Incubated | Control | Incubated | Control | Incubated |
| B1 | 88.1± 6.50 | 49.3± 3.69 | 44.5± 2.33 | 70.1 ± 5.11 | 52.56 ± 2.93 | 35.2± 2.14 | 79.3± 4.94 | 101.6± 7.62 | 21.8± 1.53 | 18.3 ± 1.63 |
| B2 | 17.8± 1.23 | 8.72 ± 0.65 | 6.74 ± 0.41 | 9.46 ± 0.61 | 3.77 ± 0.27 | 1.61 ± 0.13 | 5.03 ± 0.25 | 9.84 ± 0.63 | 2.78 ± 0.18 | 3.05 ± 0.27 |
| B3 | 1397± 103.7 | 546.0± 40.25 | 398.1± 28.74 | 562.1 ± 39.15 | 575.5 ± 43.18 | 374.0 ± 27.04 | 388.9 ± 29.15 | 498.6± 36.12 | 126 ± 8.43 | 130.4 ± 9.32 |
| B4 | 157.6± 10.81 | 87.2 ± 3.51 | 34.2± 2.14 | 85.1± 5.24 | 315.2 ± 19.62 | 208.9± 14.63 | 18.0 ± 1.45 | 41.2 ± 3.09 | 8.71 ± 0.43 | 10.3 ± 0.93 |
| B5 | 1265± 90.85 | 552.3 ± 32.69 | 502.6 ± 36.68 | 767.8± 52.12 | 810.8 ± 60.25 | 612.3 ± 45.98 | 177.2 ± 13.28 | 232.6 ± 16.43 | 67.1 ± 4.48 | 71.4 ± 5.43 |
| B6 | 261.8± 18.36 | 130 ± 9.72 | 84.2± 6.31 | 169.1± 10.74 | 214.3 ± 15.25 | 153.6 ± 10.15 | 61.1 ± 3.21 | 78.4 ± 5.87 | 25.7 ± 1.83 | 26.1 ± 1.89 |
| B7 | 739.0± 55.36 | 402.1 ± 29.25 | 442.3 ± 33.15 | 776.3± 51.36 | 378.5 ± 27.35 | 238.8 ± 15.90 | 288.3 ± 22.61 | 413.1 ± 29.09 | 181.9 ± 12.64 | 187.3 ± 14.21 |
| B8 | 156.0± 10.70 | 85.1 ± 6.25 | 123 ± 8.26 | 173.4± 12.84 | 55.6 ± 3.29 | 35.2 ± 2.41 | 33.0 ± 2.35 | 62.8 ± 4.21 | 25.0 ± 1.87 | 24.71 ± 1.98 |
| B9 | 2817± 211.1 | 1264± 92.36 | 778.3± 50.31 | 961.4 ± 71.36 | 1298 ± 91.36 | 819.6 ± 61.91 | 544.2 ± 42.79 | 697.7 ± 53.31 | 334.9 ± 26.10 | 342.8 ± 25.96 |
| B10 | 2032 ± 152.3 | 924.8± 58.69 | 203.2± 15.19 | 303.2± 19.72 | 1220± 88.41 | 1117± 84.79 | 388.3 ± 28.11 | 523.7± 38.14 | 157.3 ± 12.89 | 163.5 ± 13.26 |

**Table S13**. Geochemical fractionation of Pb in Detroit soils after 7 days of incubation (mg/kg). Values are mean ± SD (n = 3).

| **Sample ID** | **F1 – Soluble + Exchangeable** | | **F2 – Carbonate-bound** | | **F3 – Oxide-bound** | | **F4 – Organic Matter** | | **F5 – Silicate-bound** | |
| --- | --- | --- | --- | --- | --- | --- | --- | --- | --- | --- |
|  | Control | Incubated | Control | Incubated | Control | Incubated | Control | Incubated | Control | Incubated |
| D1 | 42 ± 2.9 | 37 ± 2.4 | 55 ± 3.8 | 61± 4.1 | 32 ± 2.7 | 53± 3.6 | 144± 10.2 | 129± 8.80 | 80± 5.8 | 81± 5.6 |
| D2 | 32 ± 2.2 | 27± 1.7 | 139± 9.20 | 154± 10.1 | 245± 18.4 | 279± 19.3 | 196± 13.5 | 178± 12.2 | 49± 3.9 | 50± 4.1 |
| D3 | 15 ± 1.1 | 11 ± 0.9 | 30 ± 2.3 | 39± 2.6 | 84 ± 5.5 | 99± 6.9 | 401± 28.3 | 382± 25.2 | 45± 3.1 | 44 ± 3.2 |
| D4 | 21± 1.6 | 18 ± 1.3 | 22± 1.5 | 24± 1.7 | 9.8 ± 0.7 | 12± 1.0 | 55 ± 3.9 | 50± 3.4 | 17± 1.3 | 18 ± 1.5 |
| D5 | 41± 2.8 | 34 ± 2.3 | 279± 19.7 | 301± 20.1 | 432± 29.8 | 468± 30.8 | 721± 51.4 | 692± 45.3 | 65± 4.5 | 67± 4.6 |
| D6 | 37± 2.6 | 30± 2.3 | 251± 17.3 | 287± 18.9 | 391± 27.7 | 416± 28.2 | 315± 21.7 | 294± 20.1 | 56± 4.0 | 57 ± 4.2 |
| D7 | 40± 2.9 | 36 ± 2.6 | 85± 6.1 | 91± 6.1 | 62 ± 4.8 | 73± 5.1 | 377± 26.4 | 363± 23.9 | 95± 7.1 | 96 ± 7.3 |
| D8 | 51± 3.7 | 44± 3.4 | 28± 2.0 | 32± 3.2 | 132± 9.80 | 148± 10.2 | 181± 12.4 | 169± 12.0 | 28 ± 2.1 | 27± 2.1 |
| D9 | 41 ± 2.9 | 35 ± 2.4 | 54± 3.9 | 60± 4.2 | 52± 3.8 | 65± 4.4 | 231± 16.9 | 215± 15.3 | 16± 1.2 | 17± 1.1 |
| D10 | 43± 3.1 | 37 ± 2.4 | 355± 25.8 | 374± 24.9 | 361± 24.2 | 383± 25.1 | 308± 22.3 | 282± 20.3 | 47 ± 3.5 | 48± 3.2 |

**Table S14**. Geochemical fractionation of Pb in Detroit soils after 30 days of incubation (mg/kg). Values are mean ± SD (n = 3).

| **Sample ID** | **F1 – Soluble + Exchangeable** | | **F2 – Carbonate-bound** | | **F3 – Oxide-bound** | | **F4 – Organic Matter** | | **F5 – Silicate-bound** | |
| --- | --- | --- | --- | --- | --- | --- | --- | --- | --- | --- |
|  | Control | Incubated | Control | Incubated | Control | Incubated | Control | Incubated | Control | Incubated |
| D1 | 42.2±1.35 | 34.1±1.25 | 59.2±2.41 | 67.4±2.54 | 39.6±2.07 | 63.5±4.01 | 188.1±14.51 | 129±8.40 | 32±3.0 | 34±0.61 |
| D2 | 30.1±0.12 | 25±0.12 | 146.9±7.05 | 157±6.01 | 274.1±14.92 | 299.4±19.42 | 196.0±15.12 | 168.4±11.06 | 30.1±2.74 | 31.8±0.27 |
| D3 | 13±0.41 | 10±0.48 | 35.6±1.78 | 40.1±1.62 | 63.6±3.38 | 106±6.61 | 446.2±34.61 | 360.2±23.45 | 27.4±2.51 | 28.9±0.26 |
| D4 | 20.2±0.89 | 17.1±0.75 | 29.5±2.4 | 25.3±1.11 | 8.15±0.34 | 13.2±0.91 | 45.2±3.37 | 47.5±3.11 | 18.3±1.67 | 19.4±0.77 |
| D5 | 38.8±2.23 | 32±0.36 | 286.5±12.11 | 307.8±12.14 | 393.6±21.47 | 502.0±30.82 | 813.0±63.28 | 652.4±42.48 | 38.1±3.47 | 40.3±0.16 |
| D6 | 34.6±2.05 | 29.1±2.04 | 263.2±11.91 | 294.1±11.12 | 380.1±20.73 | 446.9±27.44 | 375.2±29.18 | 277.6±19.02 | 34.1±3.11 | 36.0±0.21 |
| D7 | 36.5±2.19 | 34.0±1.89 | 90.3±3.89 | 93.1±3.12 | 41.6±2.18 | 80.6±5.23 | 449.2±34.85 | 342.8±22.41 | 42±3.9 | 45.3±0.42 |
| D8 | 50.1±3.73 | 41.8±2.65 | 26±2.1 | 33.4±1.34 | 138±7.50 | 159.1±8.76 | 190.8±14.72 | 159.4±10.42 | 14.3±1.31 | 15.2±0.22 |
| D9 | 39.6±2.36 | 32.1±2.71 | 51.0±3.44 | 62.1±2.47 | 28.1±1.44 | 70±4.3 | 258±20.1 | 202.9±13.08 | 9.79±0.92 | 10.35±0.17 |
| D10 | 39.9±1.31 | 34.9±1.27 | 357.7±15.73 | 382.1±14.45 | 381±20.8 | 411.5±25.27 | 299.8±23.28 | 265.9±17.44 | 35.0±3.21 | 37.0±0.21 |

**Table S15**. Geochemical fractionation of Pb in Detroit soils after 90 days of incubation (mg/kg). Values are mean ± SD (n = 3).

| **Sample ID** | **F1 – Soluble + Exchangeable** | | **F2 – Carbonate-bound** | | **F3 – Oxide-bound** | | **F4 – Organic Matter** | | **F5 – Silicate-bound** | |
| --- | --- | --- | --- | --- | --- | --- | --- | --- | --- | --- |
|  | Control | Incubated | Control | Incubated | Control | Incubated | Control | Incubated | Control | Incubated |
| D1 | 40.5±1.35 | 29.9±1.41 | 62.1±2.28 | 76.0±2.70 | 42.3±2.13 | 70.6±4.01 | 186.7±14.97 | 104±7.77 | 33.4±2.85 | 34.8±0.86 |
| D2 | 29.6±0.11 | 22.7±0.13 | 142±7.13 | 176±6.87 | 273.6±16.06 | 358.0±18.74 | 199.0±13.95 | 148.2±11.02 | 31.1±2.76 | 33.1±0.27 |
| D3 | 15.0±0.44 | 9.22±0.46 | 36.1±1.79 | 43.3±1.60 | 64.0±3.23 | 127±7.17 | 448.9±32.76 | 294.9±24.11 | 25.9±2.31 | 27.8±0.27 |
| D4 | 21.0±0.84 | 14.4±0.84 | 31.8±2.31 | 26.5±1.21 | 8.78±0.31 | 15.7±0.98 | 45.2±3.11 | 40.7±3.34 | 17.3±1.73 | 18.5±0.73 |
| D5 | 34.3±2.34 | 28.7±0.39 | 291.9±11.49 | 351.7±13.17 | 392.0±20.25 | 576.9±34.55 | 803.3±58.93 | 526.4±44.55 | 37.7±3.58 | 41.7±0.15 |
| D6 | 35.0±2.10 | 26.1±1.98 | 258.8±12.02 | 327.4±11.07 | 383.7±21.57 | 494.2±29.33 | 382.5±27.44 | 242.3±21.67 | 33.9±2.90 | 36.3±0.21 |
| D7 | 37.2±2.38 | 29.0±2.08 | 89.6±3.76 | 102±3.36 | 43.9±2.26 | 89.9±5.23 | 445.2±34.04 | 287.5±24.16 | 41.8±3.93 | 43.7±0.40 |
| D8 | 47.7±3.53 | 34.6±2.96 | 26.8±2.23 | 36.8±1.21 | 139±7.13 | 187±8.13 | 193.2±13.88 | 140.6±10.56 | 16.2±1.18 | 14.9±0.20 |
| D9 | 41.1±2.50 | 25.9±2.64 | 50.8±3.35 | 67.6±2.81 | 26.8±1.33 | 79.3±3.93 | 254.8±21.78 | 182.2±12.36 | 8.88±0.88 | 9.72±0.17 |
| D10 | 42.5±1.31 | 29.2±1.31 | 364.6±14.71 | 420.1±15.74 | 376.0±22.64 | 484.8±24.21 | 304.5±24.49 | 225.6±16.96 | 34.4±3.29 | 36.3±0.20 |

**Preliminary Life Cycle Assessment Data**

Goal and Scope Definition: The goal was to quantify the cradle-to-application environmental impacts associated with immobilizing Pb in contaminated garden soils using the selected amendments, pistachio shell biochar + lime, alum, and gypsum, applied as a single field treatment. The functional unit was defined as the treatment of 1 m² of Pb-contaminated soil to a depth of 30 cm, assuming a bulk density of 1,500 kg m⁻³ (450 kg of soil). The system boundary encompassed amendment production, transportation, and field application. Field application of selected amendments was assumed mechanical or manual, and minor emissions from soil mixing disturbance were not considered, as these contributions are negligible relative to transportation impacts

Amendment doses and assumed transportation distances are presented in Table S16.

**Table S16**. Amended dose and transportation information.

| Soil (city/state) | Amendment dose per m^2^ of soil to a depth of 30 cm | Transportation distance (miles/km) |
| --- | --- | --- |
| Baltimore (Maryland) | Pistachio shell biochar: 22.5 kg  Calcium hydroxide; 0.045 kg | Pistachio shell biochar: 2695/4340  Calcium hydroxide: 150/240 |
| San Antonio (Texas) | Gypsum; 13.5 kg | 450/725 |
| Detroit (Michigan) | Detroit: 0.25 kg | 700/1126 |

**Table S17.** Amendment production inventory (relevant process only).

| Amendment | Process Description | Main Inputs & Emissions | Data Source |
| --- | --- | --- | --- |
| Biochar (pistachio shell) | Pyrolysis at 500 °C with 30 % yield; produced in California | Energy: 2.5-3.2 MJ kg⁻¹ biochar (natural gas + electricity). Emissions: 0.12-0.18 kg CO₂e kg⁻¹ biochar; minor CO, CH₄, and particulates. | Ecoinvent 3.9 “Biochar production, wood chips, slow pyrolysis” |
| Alum (Al₂(SO₄)₃·14H₂O) | From bauxite via sulfuric acid digestion | Energy: 4.5-6.0 MJ kg⁻¹. Emissions: 0.10-0.15 kg CO₂e kg⁻¹; releases SO₂, fluoride, and acid mist. | Ecoinvent 3.9  “Aluminum sulfate production” |
| Gypsum  (CaSO₄·2H₂O) | Mined and  crushed natural gypsum | Energy: 0.1-0.3 MJ kg^-1^. Emissions: 0.05-0.07 kg CO₂e kg⁻¹; dust and minor SO₂/NOₓ from quarry equipment. | Ecoinvent 3.9  “Gypsum, mineral at mine” |
| Calcium  Hydroxide (Ca(OH)₂) | From quicklime hydration | Energy: 1.0-1.5 MJ kg⁻¹. Emissions: 0.10-0.15 kg CO₂e kg⁻¹; process emits CO₂ from CaCO₃ calcination. | Ecoinvent 3.9 “Calcium hydroxide production” |

**Table S18.** Transportation emission factors (GREET 2022 and EPA MOVES, 2023

| Pollutant | Emission Factor |
| --- | --- |
| CO₂ | 2.68 kg per L diesel |
| CH₄ | 0.005–0.01 g per ton·km |
| N₂O | 0.003–0.006 g per ton·km |
| NOₓ | 0.6–1.1 g per ton·km |
| SO₂ | 0.04–0.08 g per ton·km (ULSD sulfur content) |
| PM₂.₅ | 0.0010–0.0014 g per ton·km |

Assuming a diesel fuel intensity of 0.026 L per ton-km, a diesel energy content of 37 MJ per liter, and using the amendment weights and transportation distances listed in Table S16, the following transportation inventory analysis for the three types of amendments was developed and presented in Table S19.

**Table S19.** Transportation Inventory analysis per functional unit (FU, treatment of 1m^2^ of soil to a depth of 30 cm). site

| Site | Mass Transported (kg) | Diesel Use (L) | Energy (MJ) | CO₂ (kg) | NOₓ (g) | SO₂ (g) | PM₂.₅ (g) | CH₄ (g) | N₂O (g) |
| --- | --- | --- | --- | --- | --- | --- | --- | --- | --- |

| Detroit | 0.25 | 0.0073 | 0.26 | 0.02 | 0.24 | 0.017 | 0.00034 | 0.0021 | 0.0013 |
| --- | --- | --- | --- | --- | --- | --- | --- | --- | --- |

| San Antonio | 13.5 | 0.25 | 9.2 | 0.68 | 8.3 | 0.59 | 0.0117 | 0.073 | 0.044 |
| --- | --- | --- | --- | --- | --- | --- | --- | --- | --- |

| Baltimore | 22.5 (biochar) +  0.045 (Ca(OH)₂) | 2.54 | 91.4 | 6.8 | 83 | 5.9 | 0.117 | 0.73 | 0.44 |
| --- | --- | --- | --- | --- | --- | --- | --- | --- | --- |

**Table S20.** Total energy consumption per FU (transportation + amendment production)

| Site | Amendment Production Energy (MJ/m²) | Transport Energy (MJ/m²) | Total Energy (MJ/m²) |
| --- | --- | --- | --- |

| Detroit Alum | 1.1–1.5 | 0.26 | 1.36–1.76 |
| --- | --- | --- | --- |

| San Antonio Gypsum | 1.4–4.1 | 9.2 | 10.6–13.3 |
| --- | --- | --- | --- |

| Baltimore Biochar +Ca(OH)₂ | 56–72 | 91.4 | 147–163 |
| --- | --- | --- | --- |

In order to compare the proposed immobilization approach with the soil excavation-landfilling alternative the total diesel consumption (excavation + 50 miles transportation) were computed and presented in Tables S21. Furthermore, the emission factors presented in Table S18 were applied to the data in Table S21 to estimate air emissions and burden to other environmental categories following ReCiPe 2016 H model.

**Table S21.** Estimation of the total diesel consumption per FU for the excavation-landfilling option

| Process Step | Mass /Volume | Distance (km) | t·km | Diesel Use (L) |
| --- | --- | --- | --- | --- |

| Soil excavation (0.3 m³) | 0.3 m³ | – | – | 0.09–0.18 |
| --- | --- | --- | --- | --- |

| Soil transport | 0.45 t | 80 km | 36 | 0.94 |
| --- | --- | --- | --- | --- |

| Total diesel | – | – | – | 1.03–1.12 |
| --- | --- | --- | --- | --- |
